# MiR-182-5p and its target HOXA9 in non-small cell lung cancer: a clinical and in-silico exploration with the combination of RT-qPCR, miRNA-seq and miRNA-chip

**DOI:** 10.1186/s12920-019-0648-7

**Published:** 2020-01-06

**Authors:** Li Gao, Shi-bai Yan, Jie Yang, Jin-liang Kong, Ke Shi, Fu-chao Ma, Lin-zhen Huang, Jie Luo, Shu-ya Yin, Rong-quan He, Xiao-hua Hu, Gang Chen

**Affiliations:** 1 Department of Pathology, the First Affiliated Hospital of Guangxi Medical University, Zhuang Autonomous Region, Nanning, 530021 Guangxi China; 2 Department of Medical Oncology, the First Affiliated Hospital of Guangxi Medical University, Zhuang Autonomous Region, Nanning, 530021 Guangxi China; 3 Department of Pharmacology, School of Pharmacy, Guangxi Medical University, Zhuang Autonomous Region, Nanning, 530021 Guangxi China; 4 Department of Respiratory and Critical Care Medicine, the First Affiliated Hospital of Guangxi Medical University, Zhuang Autonomous Region, Nanning, 530021 Guangxi China; 5 Department of Medical Oncology, the Second Affiliated Hospital of Guangxi Medical University, Zhuang Autonomous Region, Nanning, 530021 Guangxi China

**Keywords:** miR-182-5p, Non-small cell lung cancer, RT-qPCR, miRNA-seq, miRNA-chips, HOXA9

## Abstract

**Background:**

MiR-182-5p, a cancer-related microRNA (miRNA), modulates tumorigenesis and patient outcomes in various human malignances. This study interroted the clinicopathological significance and molecular mechanisms of miR-182-5p in non-small cell lung cancer (NSCLC).

**Methods:**

The clinical significance of miR-182-5p in NSCLC subtypes was determined based on an analysis of 124 samples (lung adenocarcinomas [LUADs], *n* = 101; lung squamous cell carcinomas [LUSCs], *n* = 23) obtained from NSCLC patients and paired noncancer tissues and an analysis of data obtained from public miRNA-seq database, miRNA-chip database, and the scientific literature. The NSCLC samples (*n* = 124) were analyzed using the real-time quantitative polymerase chain reaction (RT-qPCR). Potential targets of miR-182-5p were identified using lists generated by miRWalk v.2.0, a comprehensive atlas of predicted and validated targets of miRNA-target interactions. Molecular events of miR-182-5p in NSCLC were unveiled based on a functional analysis of candidate targets. The association of miR-182-5p with one of the candidate target genes, homeobox A9 (*HOXA9*), was validated using in-house RT-qPCR and dual-luciferase reporter assays.

**Results:**

The results of the in-house RT-qPCR assays analysis of data obtained from public miRNA-seq databases, miRNA-chip databases, and the scientific literature all supported upregulation of the expression level of miR-182-5p level in NSCLC. Moreover, the in-house RT-qPCR data supported the influence of upregulated miR-182-5p on malignant progression of NSCLC. In total, 774 prospective targets of miR-182-5p were identified. These targets were mainly clustered in pathways associated with biological processes, such as axonogenesis, axonal development, and Ras protein signal transduction, as well as pathways involved in axonal guidance, melanogenesis, and longevity regulation, in multiple species. Correlation analysis of the in-house RT-qPCR data and dual-luciferase reporter assays confirmed that *HOXA9* was a direct target of miR-182-5p in NSCLC.

**Conclusions:**

The miR-182-5p expression level was upregulated in NSCLC tissues. MiR-182-5p may exert oncogenic influence on NSCLC through regulating target genes such as HOXA9.

## Background

According to data from the National Comprehensive Cancer Network, lung cancer (LC) is responsible for the majority of cancer-associated deaths worldwide [1]. There are two types of LC: non-small cell lung cancer (NSCLC) and small cell lung cancer [2]. Of these, NSLC is the most common and accounts for the majority cases of LC [1–8].

Although improvements in screening (i.e., diagnostic imaging and laboratory tests) and drug therapy have contributed greatly to NSCLC outcomes, the clinical outcome of NSCLC remains poor due to a lack of effective biomarkers for NSCLC [4]. At the time of diagnosis, most of patients have advanced stage disease because of atypical symptoms in the early stage of the disease [2]. Thus, NSCLC survival is poor, with 5-year survival lower than 20% [5]. Therefore, the development of novel screening and therapeutic strategies are of crucial importance for NSCLC patients.

MicroRNAs (miRNAs) are small, noncoding RNAs that regulate gene expression by binding specifically to the complimentary sequence of target mRNAs in the 3′ untranslated region (3′-UTR), thereby silencing the translation process and accelerating the degradation of target mRNAs [5, 8–16]. A number of previous studies demonstrated that miRNAs played essential roles in various cancers, including LC, via their effects on various biological events, such as differentiation, apoptosis, and proliferation, at the post-transcriptional level [9, 17–24]. Studies also reported that the miRNA miR-182-5p participated in the occurrence and progression of various human cancers [25–30]. In previous work, we demonstrated a tumor-promoting effect of upregulated miR-182-5p in lung squamous cell carcinomas (LUSCs) [31].

The aim of the present study was to examine the clinicopathological value and molecular mechanisms of miR-182-5p in non-small cell lung cancer (NSCLC). With this aim in mind, we examined miR-182-5p overexpression patterns in lung adenocarcinomas (LUADs) and NSCLC.

We expect that the current study will facilitate understanding of the role of miR-182-5p in the pathogenesis of NSCLC and its potential value as a marker of NSCLC subtypes.

## Methods

### Tissue collection from NSCLC patients

NSCLC tissue samples and paired noncancer tissue samples were obtained from 124 NSCLS patients (LUADs, *n* = 101; LUSCs, *n* = 23) undergoing surgery at the First Affiliated Hospital of Guangxi Medical University between January 2012 and February 2014. All the NSCLC cases were diagnosed by two independent pathologists with no involvement in the study. There were 74 males and 50 females in this study.

The study was approved by the ethics committee of the First Affiliated Hospital of Guangxi Medical University, and written informed consent was obtained from all the patients.

All 124 NSCLC tissues were formalin fixed and paraffin embedded for subsequent experiments.

### In-house real-time quantitative polymerase chain reaction (RT-qPCR)

Isolation and normalization of RNA, and the RT-qPCR were carried out as previously described [31–38]. *RNU6B* was selected as an endogenous control and reference gene of miR-182-5p [34]. The sequences of miR-182-5p and *RNU6B* were as follows: miR-182-5p: UUUGGCAAUGGUAGAACUCACACU (Cat. no. 4427975–002334); *RNU6B*: CGCAAGGAUGACACGCAAAUUCGUGAAGCGUUCCAUAUUUUU (Cat. no. 4427975–000490). The relative miR-182-5p expression level was computed using the method of 2^-Δcq^. The statistical analysis for the RT-qPCR was as described previously [39].

### MiR-182-5p expression in NSCLC using miRNA-seq data

Level 3 IlluminaHiSeq miRNA-seq data on miR-182-5p expression in NSCLC were obtained from recomputed and normalized the cancer genome atlas (TCGA) data in UCSC Xena (https://xena.ucsc.edu/). Alterations in the expression of miR-182-5p in LUADs, non-LUADs, NSCLC, and non-NSCLC, in addition to the distribution of miR-182-5p in groups of different clinical variables, were calculated to determine the clinical significance of miR-182-5p. The statistical analysis of the miRNA-seq data has been described in detail elsewhere [39].

### Analysis of miR-182-5p expression in NSCLC based on data in public miRNA-chip databases

The gene expression omnibus (GEO) database was searched for data deposited up to 12 April 2019. The search terms used were as follows: (cancer OR carcinoma OR adenocarcinoma OR tumour OR tumor OR malignanc* OR neoplas*) AND (lung OR pulmonary OR respiratory OR respiration OR aspiration OR bronchi OR bronchioles OR alveoli OR pneumocytes OR “air way”). All miRNA-chips meeting the listed inclusion criteria were considered eligible for inclusion in the study: (1) the experimental subjects were humans, and (2) miR-182-5p expression was reported for both NSCLC and healthy lung tissues. Only studies where the NSCLC sample size exceeded three and included paired healthy tissues were included. The data extraction and statistical analysis of miR-182-5p expression have been described in detail elsewhere [40].

### Search of the scientific literature for data on miR-182-5p expression in NSCLC

We searched the literature for information of miR-182-5p differential expression in NSCLC and noncancer lung tissues. The following databases were searched using the keywords: (cancer OR carcinoma OR adenocarcinoma OR tumour OR tumor OR malignanc* OR neoplas*) AND (Lung OR pulmonary OR respiratory OR respiration OR aspiration OR bronchi OR bronchioles OR alveoli OR pneumocytes OR “air way”) AND (miR-182 OR miRNA-182 OR microRNA-182 OR miR182 OR miRNA182 OR microRNA182 OR “miR 182” OR “miRNA 182” OR “microRNA 182”OR miR-182-5p OR miRNA-182-5p OR microRNA-182-5p): PubMed, Wiley Online Library, EBSCO, Cochrane Central Register of Controlled Trials, Web of Science, Google Scholar, Ovid, EMBASE, and LILACS. Studies that reported expression data for miR-182-5p in NSCLC subtypes and paired noncancer samples were included in a meta-analysis. The meta-analysis included all the in-house RT-qPCR, miRNA-seq, miRNA-chip, and literature data. Pooling of the standard mean difference (SMD) and creation of summary receiver operating characteristic (SROC) curves from all the included studies was done to determine the differential expression miR-182-5p and its potential utility in distinguishing NSCLC and noncancer cases. Details on the data processing and statistical analysis in the meta-analysis have been described in previous studies [40, 41].

### Prediction of target genes

An online program, MiRWalk v.2.0, which incorporates 12 prediction platforms (miRanda, Microt4, miRWalk, miRDB, miRbridge, miRMap, Pictar2, miRNAMap, PITA, RNAhybrid, RNA22, and Targetscan), was used for predicting the targets of miR-182-5p. Predicted genes that appeared in at least eight of the 12 prediction platforms were regarded as candidate targets of miR-182-5p.

### Functional annotation of candidate target genes of miR-182-5p and construction of a protein–protein interaction (PPI) network

Gene ontology (GO) and Kyoto Encyclopedia of Genes and Genomes (KEGG) pathway analyses were conducted using the ClusterProfiler package in R software v.3.5.2 to explore the enrichment of candidate target genes in biological process, cellular component, and molecular function pathways. Items with a *p* < 0.05 were considered statistically significant. The top 15 significant biological process, cellular component, and molecular function terms, as well as the top 10 significant KEGG pathway terms, were visualized as a bubble plot and chord plot using the GOplot package of R software v.3.5.2. A PPI network was subsequently built using the Search Tool for the Retrieval of Interacting Genes to illustrate the interactions between target genes.

### Validation of miR-182-5p targeting of *HOXA9*

In a previous study, we detected the expression level of *HOXA9* in the same cohort of NSCLC patients using the RT-qPCR [42]. The primers for *HOXA9* and the internal control: glyceraldehyde-3-phosphate dehydrogenase (*GAPDH*) were as follows: 5′-GCTGAGAATGAGAGCGGC-3′ (*HOXA9* forward); 5′-CAGTTCCAGGGTCTGGTGTT-3′ (*HOXA9* reverse); 5-′TGCACCACCAACTGCTTA-3′ (*GAPDH* forward); and 5′-GGATGCAGGGATGATGTTC-3′ (*GAPDH* reverse). The student’s paired *t* test in SPSS v.22.0 was performed to compare the expression levels of *HOXA9* and miR-182-5p. The correlation between *HOXA9* and miR-182-5p expression was assessed using Pearson’s correlation test in GraphpadPrism v.7.0.

Data on predictive binding sites between *HOXA9* and miR-182-5p were obtained from TargetScanHuman v.7.2. A dual luciferase reporter assay was performed to validate the direct target binding between *HOXA9* and miR-182-5p. The 3’UTR of *HOXA9* (wild type or mutation type) comprising putative miR-182-5p binding sites was cloned into a psiCHECK-2 luciferase reporter vector (Promega, USA) to generate psiCHECK-HOXA9 3′-UTRs or psiCHECK-HOXA9-mut 3′UTRs. HEK-293 T cells were co-transfected with an miR-182-5p mimic, a negative mimic control, and a reporter vector of the psiCHECK-HOXA9 3′UTR or psiCHECK-HOXA9-mut 3′UTR. After incubation for 27 h, the luciferase activity was measured using dual luciferase assay (Promega, USA) according to the manufacturer’s protocol. Luciferase activity was inferred based on the ratios of Renilla and firefly luciferase activity. Each experiment was repeated three times.

## Results

### Evaluation of the clinicopathological significance of miR-182-5p in NSCLC

#### Analysis of RT-qPCR data

The analysis of the RT-qPCR data demonstrated that miR-182-5p was significantly upregulated in LUAD tissues as compared with that in paired non-LUAD lung tissues (*P* < 0.001, Table 1, Additional file 1: Fig. S1). In general, miR-182-5p expression level was markedly higher in the majority of NSCLC tissues than in paired noncancer tissues (*P* < 0.001, Table 2, Additional file 2: Fig. S2). Overexpression of miR-182-5p in LUAD and NSCLC was strongly associated with clinical parameters including tumor size, TNM stage, and lymph node metastasis (*P* < 0.05, Tables 1 and 2). As shown by the ROC curve in Additional files 3 and 4: Fig. S3 and S4, miR-182-5p performed moderately well in differentiating LUAD from noncancer lung tissues and better in differentiating NSCLC tissues from noncancer lung tissues (area under the curve [AUC] = 0.68 and 0.82, respectively). Kaplan–Meier survival curves revealed insignificant difference in the survival outcomes of LUAD and NSCLC patients with low or high miR-182-5p expression (data not shown).

**Table 1:** MiR-182-5p expression in LUAD data from RT-qPCR

Clinicopathological parameters		n	Relevant expression of miR-182-5p (2^−ΔCq^)		
			Mean ± SD	t/F-value	*p*-value
Tissue	LUAD	101	30.371 ± 5.475	14.035	< 0.001*
	Noncancerous	101	22.908 ± 3.728		
Gender	Male	56	29.923 ± 5.567	1.150	0.253
	Female	45	28.609 ± 5.879		
Age (years)	< 60	41	30.642 ± 5.238	1.964	0.053
	≥60	60	28.446 ± 5.899		
Smoke	No	26	31.159 ± 5.435	0.602	0.550
	Yes	18	30.138 ± 5.671		
Tumor size	≤3 cm	53	25.041 ± 4.651	−13.541	< 0.001*
	> 3 cm	48	34.082 ± 1.343		
Vascular invasion	No	70	28.986 ± 5.839	−0.927	0.356
	Yes	31	30.130 ± 5.441		
TNM	I-II	44	26.730 ± 5.442	−4.377	< 0.001*
	III-IV	57	31.350 ± 5.114		
Lymph node metastasis	No	45	26.478 ± 5.243	−5.023	< 0.001*
	Yes	56	31.635 ± 5.036		
Pathological grading	I	17	27.642 ± 5.863	0.915^b^	0.404
	II	61	29.753 ± 5.713		
	III	23	29.489 ± 5.641		

LUAD: lung adenocarcinoma; SD: standard deviation

* The results were statistically significant (*P* < 0.05)

**Table 2:** MiR-182-5p expression in NSCLC data from RT-qPCR

Clinicopathological parameters		n	Relevant expression of miR-182-5p (2^−ΔCq^)		
			Mean ± SD	t/F-value	p-value
Tissue	NSCLC	124	29.615 ± 5.616	13.979	< 0.001*
	Noncancerous	124	22.859 ± 3.669		
Gender	Male	74	30.144 ± 5.497	1.278	0.204
	Female	50	28.833 ± 5.755		
Age (years)	< 60	56	30.913 ± 5.052	2.413	0.017*
	≥60	68	28.547 ± 5.864		
Smoke	No	38	31.184 ± 5.185	0.720	0.474
	Yes	29	30.237 ± 5.530		
Histological type	Adenocarcinoma	101	29.337 ± 5.717	−1.245	0.221
	Squamous carcinoma	23	30.836 ± 5.086		
Tumor size	≤3 cm	60	24.995 ± 4.570	−14.353	< 0.001*
	> 3 cm	64	33.947 ± 1.620		
Vascular invasion	No	90	29.456 ± 5.720	−0.512	0.609
	Yes	34	30.037 ± 5.391		
TNM	I-II	54	27.131 ± 5.424	−4.632	< 0.001*
	III-IV	70	31.532 ± 5.007		
Lymph node metastasis	No	56	26.621 ± 5.128	−6.095	< 0.001*
	Yes	68	32.081 ± 4.759		
Pathological grading	I	17	27.642 ± 5.863	1.246^a^	0.291
	II	77	29.853 ± 5.721		
	III	30	30.124 ± 5.131		

NSCLC: non-small cell lung cancer; SD: standard deviation

a One-way analysis of variance (ANOVA) was performed

* The results were statistically significant (*P* < 0.05)

### Analysis of miRNA-seq data

In total, miRNA-seq data were obtained for 784 NSCLC tissues (LUAD, *n* = 448; LUSC, *n* = 336) and 89 noncancer tissues. The clinicopathological significance of miR-182-5p in LUAD and NSCLC is summarized in Table 3 and Table 4, respectively. In both LUAD and NSCLC tissues, miR-182-5p expression was markedly higher as compared with that in noncancer tissues (*P* < 0.001, Additional files 1 and 2: Fig. S1 and S2, Tables 3 and 4). As shown by the ROC curves in Additional files 3 and 4: Fig. S3 and S4, miR-182-5p expression appeared to distinguish LUAD and NSCLC from noncancer tissues (AUC = 0.98 and AUC = 0.96, respectively). The Kaplan–Meier curves revealed no significant relationship between miR-182-5p expression and survival of NSCLC patients and LUAD patients (data not shown). Thus, the prognostic role of miR-182-5p in NSCLC remains unclear and needs to be studied in future work.

**Table 3:** MiR-182-5p expression in LUAD from miRNA-seq data

Characteristics		n	Relevant expression of miR-182-5p (log_2_x)		
			Mean ± SD	t/F-value	P-value
Tissue	LUAD	448	14.273 ± 0.947	17.368	< 0.001*
	Noncancerous	45	11.644 ± 1.160		
Gender	Male	209	14.259 ± 0.902	−0.306	0.760
	Female	239	14.286 ± 0.987		
Age(years)	≤50	33	14.295 ± 1.205	0.175	0.862
	> 50	396	14.257 ± 0.932		
T	T1 + T2	388	14.294 ± 0.954	0.679	0.497
	T3 + T4	57	14.203 ± 0.871		
Nodes	No	293	14.252 ± 0.966	−1.025	0.306
	Yes	146	14.349 ± 0.892		
Metastasis	No	285	14.326 ± 0.898	−0.366	0.715
	Yes	19	14.404 ± 1.027		
Pathologic stage	I-II	351	14.249 ± 0.958	−0.810	0.419
	III-IV	92	14.340 ± 0.918		
Anatomic neoplasm subdivision	L-Lower	70	14.229 ± 0.825	0.512^a^	0.727
	L-Upper	108	14.206 ± 0.998		
	R-Lower	85	14.214 ± 0.940		
	R-Middle	18	14.281 ± 0.789		
	R-Uppr	155	14.351 ± 0.986		
Tumor location	Peripheral	113	14.309 ± 0.940	−0.743	0.459
	Central	54	14.429 ± 1.051		

LUAD: lung adenocarcinoma; SD: standard deviation

a One-way analysis of variance (ANOVA) was performed

* The results were statistically significant (*P* < 0.05)

**Table 4:** MiR-182-5p expression in NSCLC from miRNA-seq data

Clinicopathological feature		n	Relevant expression of miR-182-5p (log_2_x)		
			Mean ± SD	t/F-value	P-value
Tissue	Lung cancer	784	14.332 ± 1.042	21.214	< 0.001*
	Noncancerous	89	11.962 ± 0.994		
Histological type	Adenocarcinoma	448	14.273 ± 0.947	−1.785	0.075
	Squamous carcinoma	336	14.411 ± 1.154		
Gender	Male	460	14.367 ± 1.042	1.137	0.256
	Female	324	14.282 ± 1.042		
Age(years)	≤60	213	14.396 ± 1.040	1.169	0.243
	> 60	546	14.297 ± 1.055		
T	T1 + T2	654	14.358 ± 1.038	1.242	0.214
	T3 + T4	127	14.232 ± 1.051		
Nodes	No	509	14.315 ± 1.050	−1.182	0.238
	Yes	260	14.408 ± 1.006		
Metastasis	No	540	14.376 ± 1.041	0.328	0.743
	Yes	21	14.302 ± 1.029		
Pathologic stage	I-II	631	14.320 ± 1.063	−0.481	0.631
	III-IV	145	14.366 ± 0.962		
Anatomic organ subdivision	L-Lower	112	14.285 ± 1.029	0.662^a^	0.618
	L-Upper	198	14.240 ± 0.995		
	R-Lower	160	14.390 ± 1.073		
	R-Middle	29	14.416 ± 1.008		
	R-Uppr	249	14.359 ± 1.026		
Tumor location	Peripheral	187	14.342 ± 1.013	−0.210	0.834
	Central	162	14.366 ± 1.130		

NSCLC: non-small cell lung cancer; SD: standard deviation

^a^ One-way analysis of variance (ANOVA) was performed

* The results were statistically significant (*P* < 0.05)

### Analysis of miRNA-chip data

**Fig. 1:**
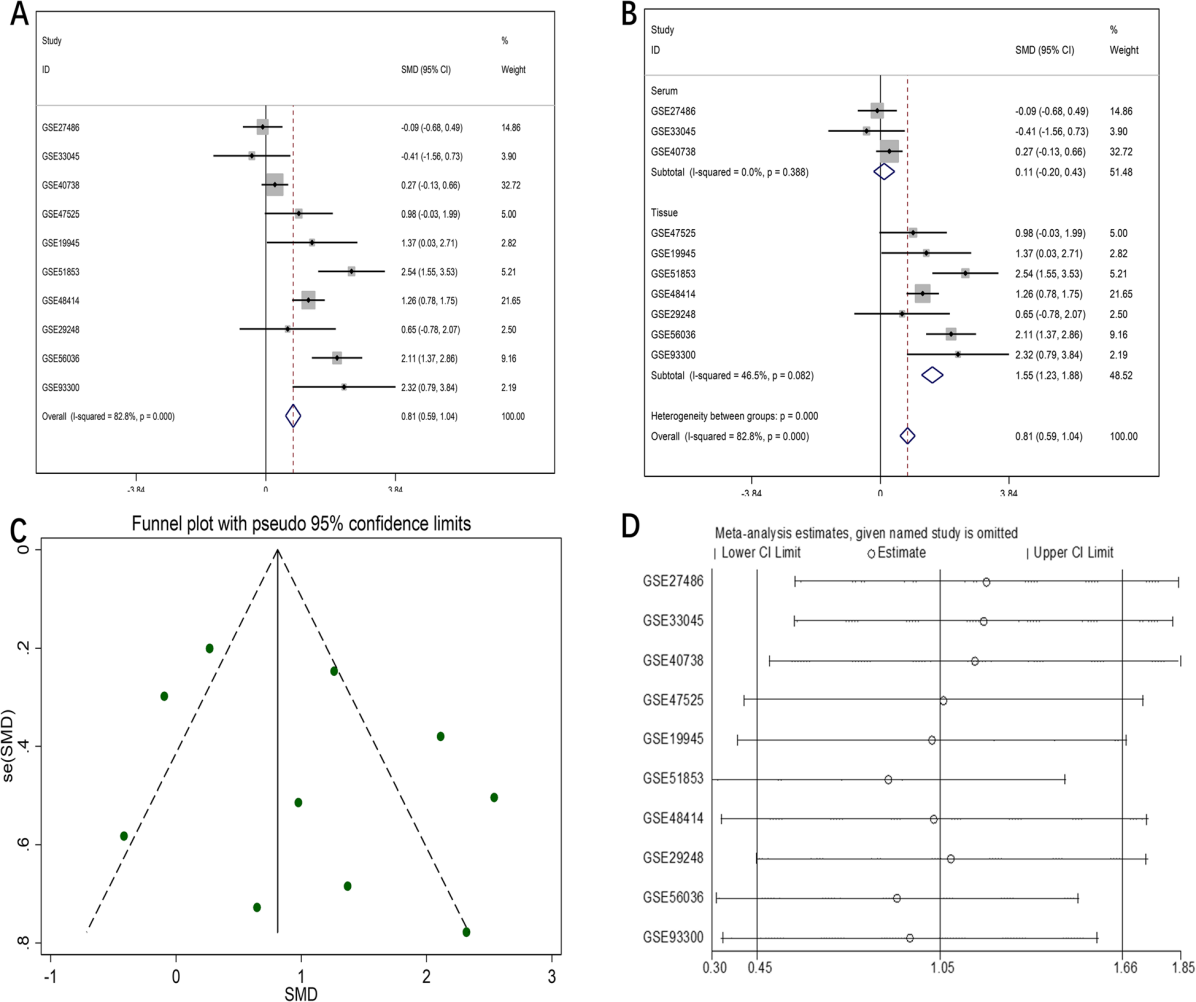
Meta-analysis of miRNA-chip data for LUAD. **a**. Forest plot for overall SMD; **b**. Subgroup analysis; **c**. Funnel plot of publication bias; **d**. Sensitivity analysis

**Fig. 2:**
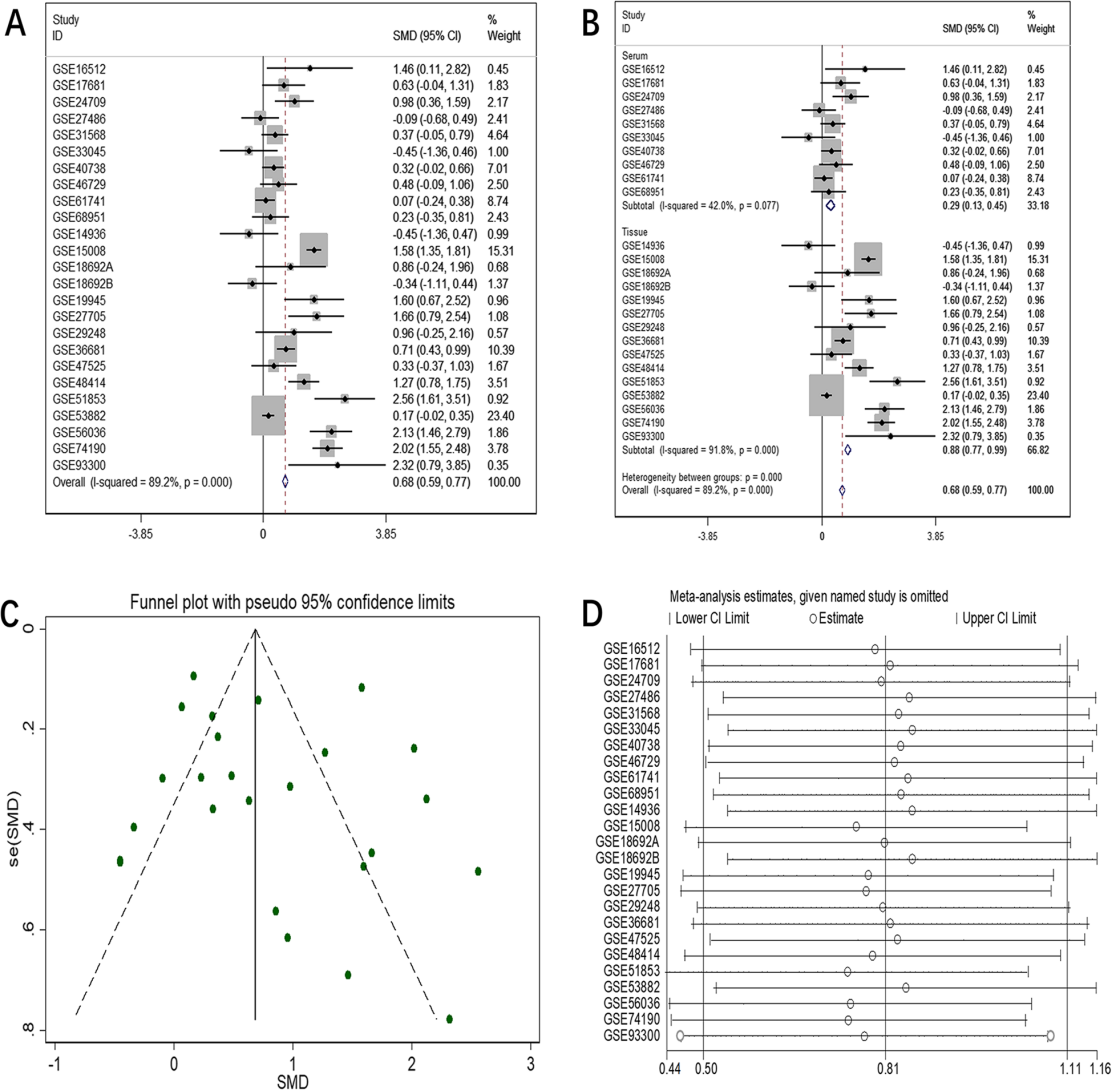
Meta-analysis of miRNA-chip data for NSCLC. **a**. Forest plot for overall SMD; **b**. Subgroup analysis; **c**. Funnel plot of publication bias; **d**. Sensitivity analysis

The initial search of the GEO database revealed 3204 studies. Of these, 248 studies were excluded after screening the titles and abstracts. The final analysis included data of 25 eligible miRNA-chips on 1656 NSCLC samples (LUAD, *n* = 350) and 948 noncancer samples. Several of the datasets that contained information on miR-182-5p expression in LUSC (GSE29248, GSE47525, GSE19945, GSE51853, and GSE74190) have been mined in previous work [31]. The characteristics of all the included miRNA-chip data are listed in Table 5. The differential expression of miR-182-5p and the discriminatory ability of miR-182-5p in distinguishing LUAD and NSCLC tissues from noncancer tissues are displayed in Additional file 1-6: Fig. S1–6. The forest plots in Figs. 1 and 2 support a noticeable increase in the miR-182-5p level in LUAD and NSCLC as compared with the level in noncancer lung samples (SMD = 0.81, 95% confidence interval [CI] = 0.59–1.04; SMD = 0.68, 95% CI = 0.59–0.77).

**Table 5:** Characteristics of included GEO miRNA-chips

	First author	Experiment type	Sample type	Platform	Cancer (N)	Cancer (M)	Cancer (SD)	Noncancer (N)	Noncancer (M)	Noncancer (SD)	TP	FP	FN	TN
GSE14936	Seike M	Non-coding RNA profiling by array	tissue	GPL8879	10	7.763	0.850	9	8.137	0.819	8	7	2	2
GSE15008	Tan X	Non-coding RNA profiling by array	tissue	GPL2009	187	11.401	1.255	188	9.697	0.867	44	1	143	187
GSE16512	Lodes MJ	Non-coding RNA profiling by array	serum	GPL8686	3	−0.024	0.031	14	−0.338	0.23	1	1	2	13
GSE17681	Keller A	miRNA Profiling	serum	GPL9040	17	12.652	0.756	19	12.174	0.753	7	1	10	18
GSE18692A	Puisségur MP	Non-coding RNA profiling by array	tissue	GPL4717	7	0.458	0.304	7	0.251	0.153	5	1	2	6
GSE18692B	Puisségur MP	Non-coding RNA profiling by array	tissue	GPL4718	13	0.288	0.343	13	0.403	0.34	10	11	3	2
GSE19945	Ohba T	Non-coding RNA profiling by array	tissue	GPL9948	20	−0.325	1.781	8	−2.799	0.554	15	0	5	8
GSE24709	Andreas Keller	Non-coding RNA profiling by array	serum	GPL9040	28	12.253	0.532	19	11.626	0.775	17	0	11	19
GSE27486	Santosh Kumar Patnaik	Non-coding RNA profiling by array	serum	GPL11432	22	5.680	0.428	23	5.731	0.646	11	10	12	13
GSE27705	Chris Fenton	Non-coding RNA profiling by array	tissue	GPL11432	20	0.106	0.934	10	−1.187	0.196	19	1	1	9
GSE29248	lina ma	Non-coding RNA profiling by array	tissue	GPL8179	6	12.643	0.704	6	11.099	2.171	3	0	3	6
GSE31568	Andreas Keller	Non-coding RNA profiling by array	serum	GPL9040	32	12.173	0.607	70	11.847	0.972	12	5	20	65
GSE33045	Sonia Molina-Pinelo	Expression profiling by RT-PCR	serum	GPL13987	12	12.618	1.642	8	13.291	1.223	12	7	0	1
GSE36681	Jin Sung Jang	Non-coding RNA profiling by array	tissue	GPL8179	103	12.151	1.215	103	11.282	1.232	16	1	87	102
GSE40738	Santosh Kumar Patnaik	Non-coding RNA profiling by array	serum	GPL16016	81	5.225	0.581	56	5.025	0.675	8	1	73	55
GSE46729	Zongli Xu	Non-coding RNA profiling by array	serum	GPL8786	24	4.530	0.227	24	4.423	0.215	5	1	19	23
GSE47525	Maikel Wouters	Non-coding RNA profiling by array	tissue	GPL17222	18	4.927	0.716	14	4.727	0.412	7	1	11	13
GSE48414	Maria Moksnes Bjaanæs	Non-coding RNA profiling by array	tissue	GPL16770	154	−0.364	2.547	20	−3.548	2.166	120	0	34	20
GSE51853	Takashi Takahashi	Non-coding RNA profiling by array	tissue	GPL7341	124	1.177	1.045	5	−1.459	0.381	120	0	4	5
GSE53882	Heng-Ying Pu	Expression profiling by array	tissue	GPL18130	397	1.245	0.446	151	1.172	0.428	11	1	386	150
GSE56036	Satoshi Kondo	Non-coding RNA profiling by array	tissue	GPL15446	28	6.397	1.319	27	4.166	0.663	25	1	3	26
GSE61741	Andreas Keller	Non-coding RNA profiling by array	serum	GPL9040	72	12.342	0.656	94	12.302	0.514	26	25	46	69
GSE68951	Christina Backes	Non-coding RNA profiling by array	serum	GPL16770	203	1.225	0.336	12	1.149	0.144	92	1	111	11
GSE74190	Lu Shaohua	Non-coding RNA profiling by array	tissue	GPL19622	66	0.761	1.650	44	−3.404	2.568	36	1	30	43
GSE93300	qu lili	Non-coding RNA profiling by array	tissue	GPL21576	9	−6.044	0.938	4	−8.187	0.885	8	0	1	4

Note: N: number; M: median; SD: standard deviation; TP: true positivity; FP: false positivity; FN: false negativity; TN: true negativity

**Fig. 3:**
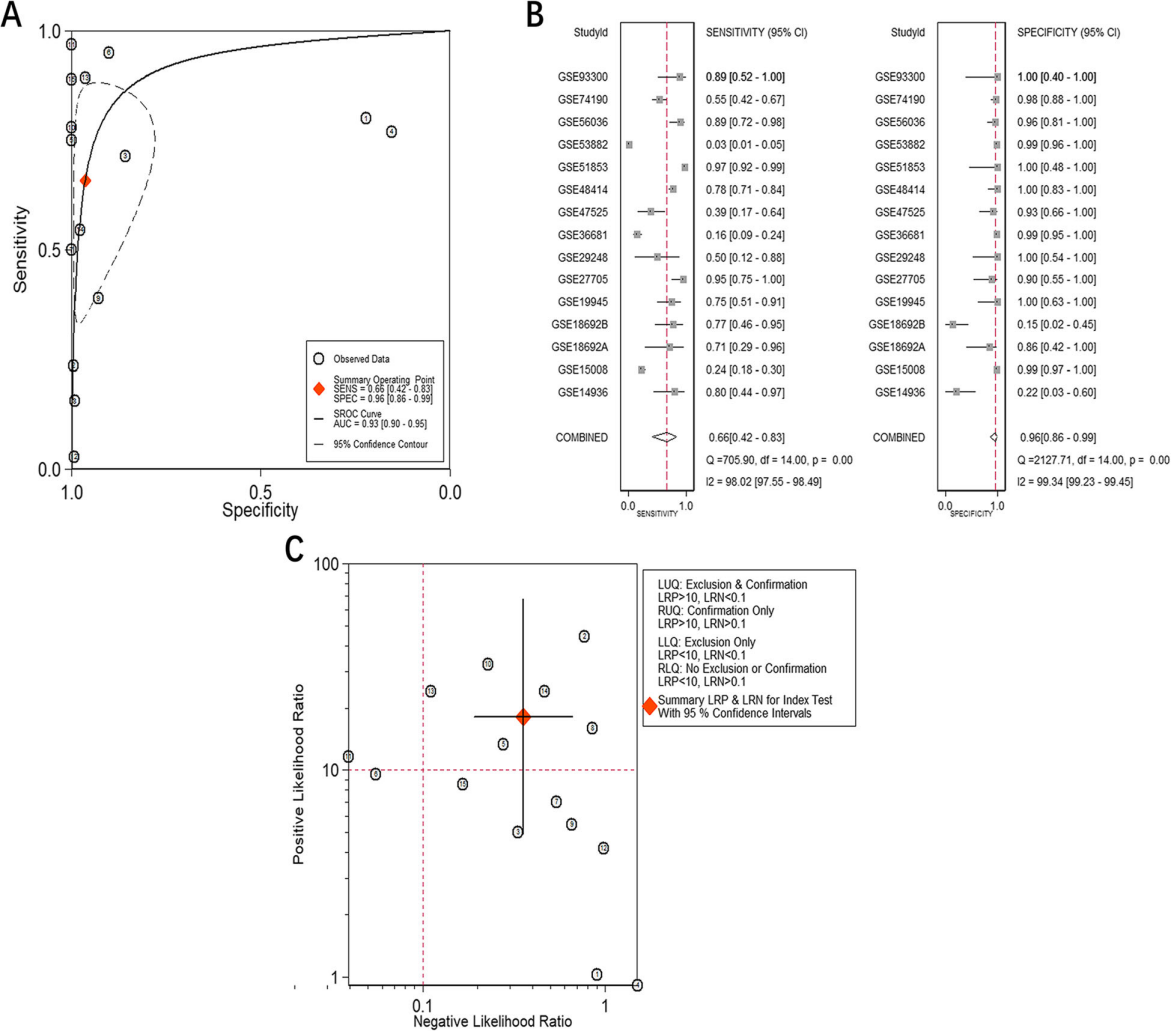
The distinguishing ability of miR-182-5p in NSCLC tissues based on data from miRNA-chips. **a**. SROC curves; **b**. Forest plot for sensitivity and specificity; **c**. Summary of positive likelihood ratio and negative likelihood ratio

**Fig. 4:**
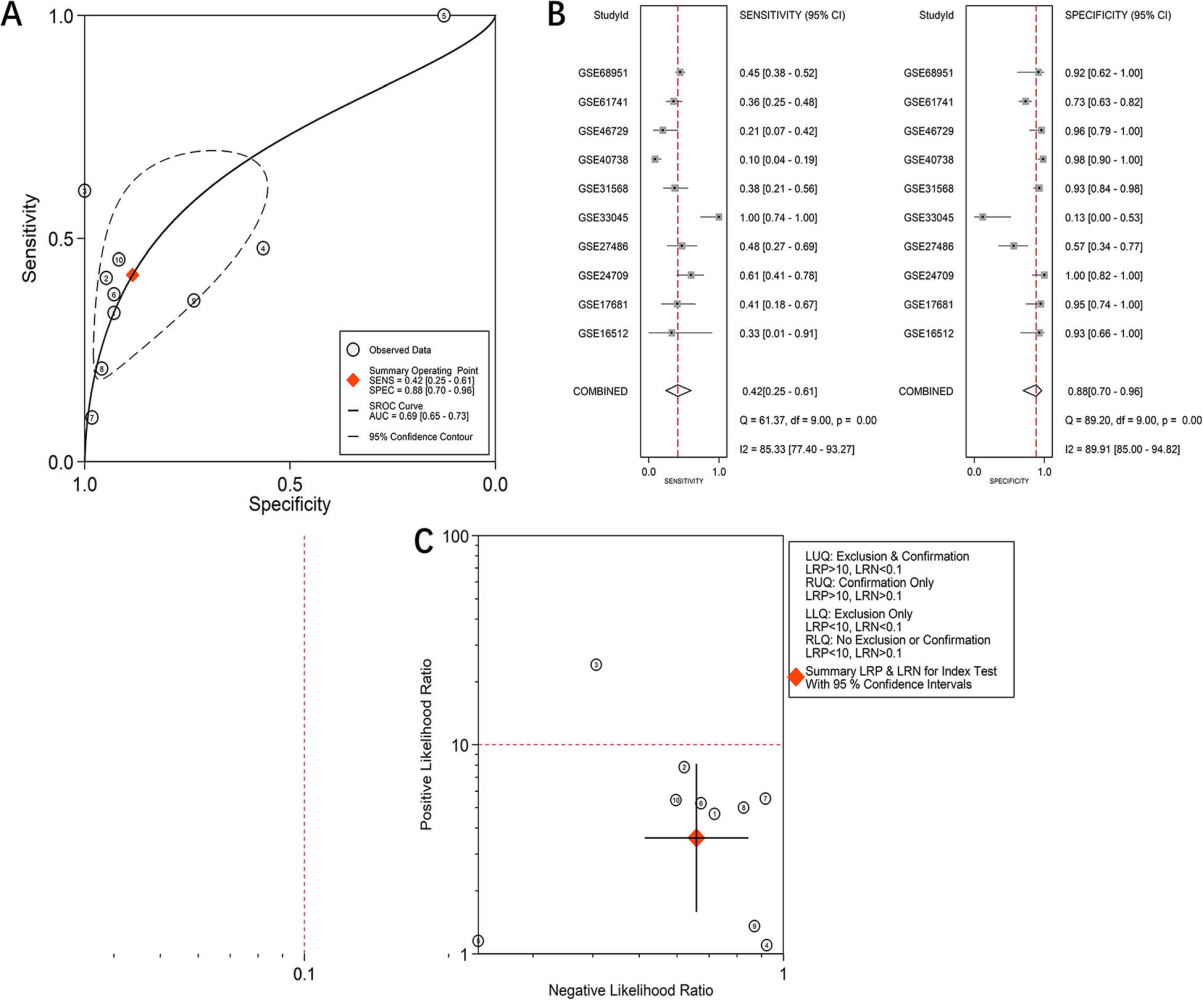
The distinguishing value of miR-182-5p in NSCLC serum based on data from miRNA-chips. **a**. SROC curves; **b**. Forest plot for sensitivity and specificity; **c**. Summary of positive likelihood ratio and negative likelihood ratio

Due to obvious heterogeneity among individual studies (I^2^ = 82.8%, *P < 0.001*; I^2^ = 89.2%, *P < 0.001*), random-effect models were applied to merge the estimates. A subgroup analysis based on the source of the samples was employed to determine the origin of the heterogeneity. The pooled SMD of miR-182-5p expression in LUAD serum and tissue samples was 0.11 (− 0.20–0.43) and 1.55 (1.23–1.88), respectively (Fig. 1). For NSCLC, the pooled SMD of miR-182-5p expression in NSCLC serum and tissue samples was 0.29 (0.13–0.45) and 0.88 (0.77–0.99), respectively, suggesting that upregulation of miR-182-5p expression was more obvious in the tissue samples than in the serum samples (Fig. 2). A subsequent sensitivity analysis and test for publication bias reported no eccentric study and no publication bias (Figs. 1 and 2). SROC curves accompanied by forest plots of the sensitivity, specificity, positive likelihood ratio, and negative likelihood ratio for NSCLC suggested that miR-182-5p expression in tissue better discriminated cancerous versus noncancerous tissue than miR-182-5p expression in serum (AUC = 0.93 and AUC = 0.69, respectively; Figs. 3 and 4).

### Results of the meta-analysis incorporating in-house RT-qPCR data, miRNA-seq data and miRNA-chip data

**Fig. 5:**
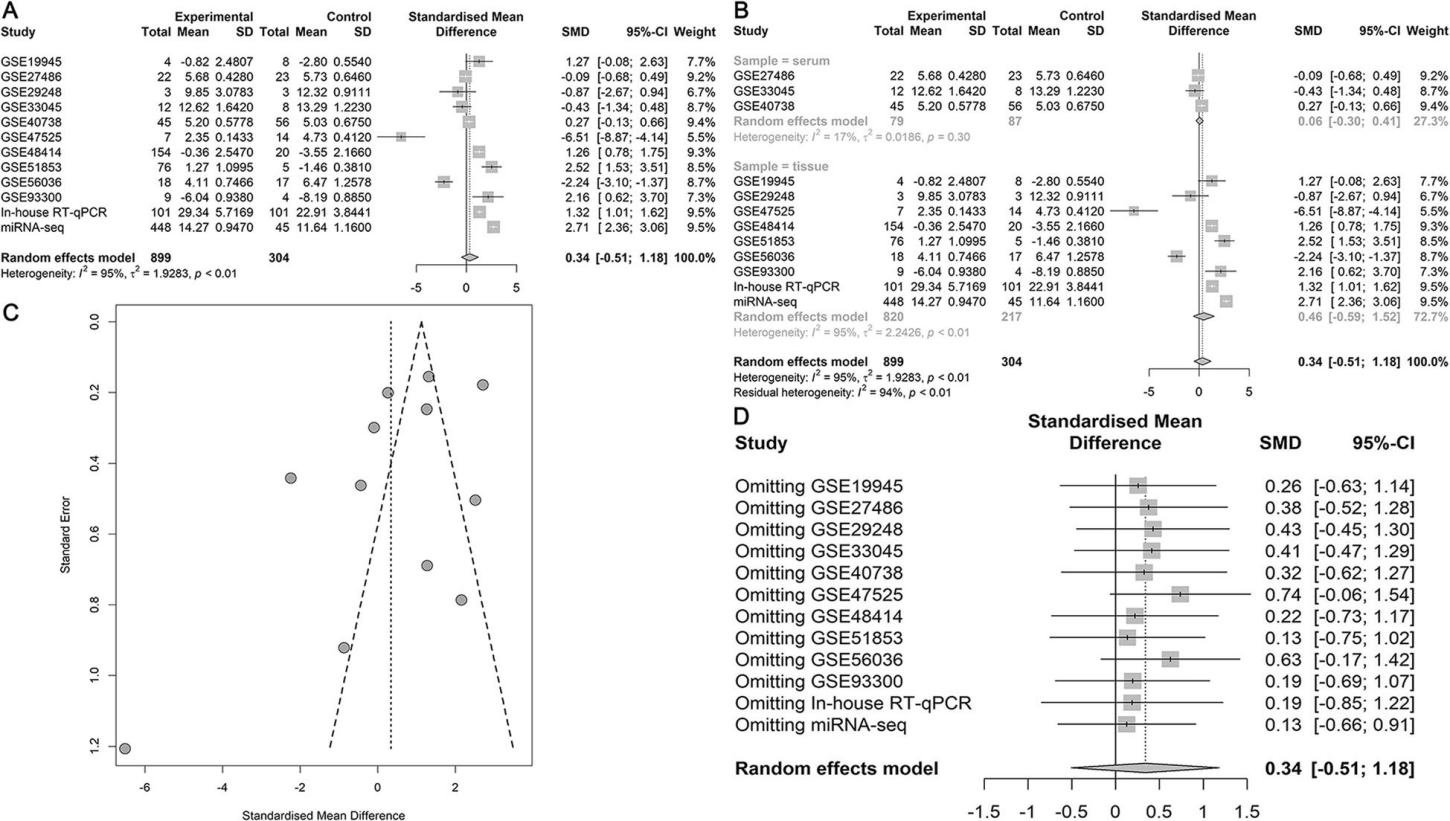
The comprehensive meta-analysis for miR-182-5p expression in LUAD. **a**. Forest plot for overall SMD; **b**. Subgroup analysis; **c**. Funnel plot of publication bias; **d**. Sensitivity analysis

**Fig. 6:**
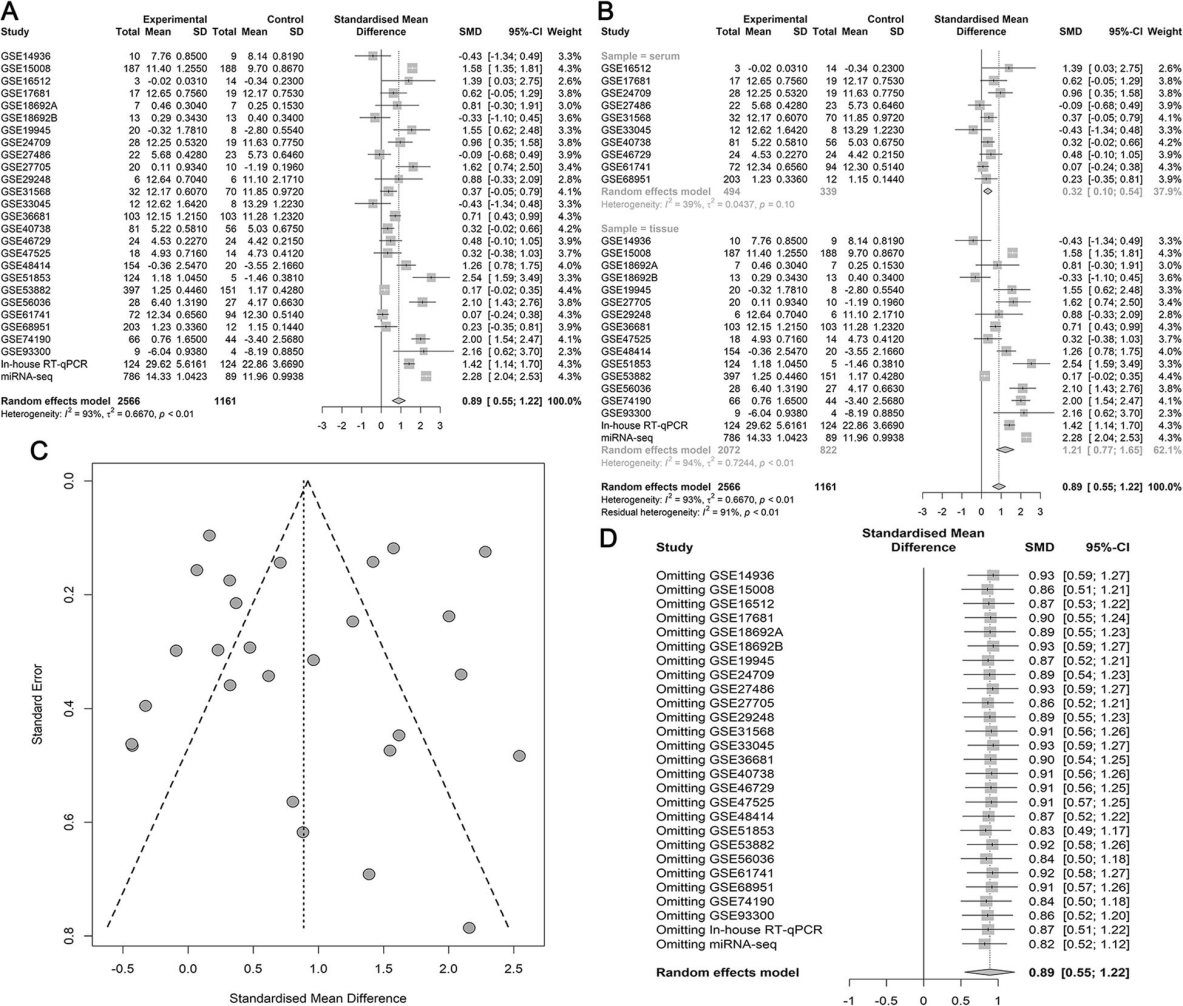
The comprehensive meta-analysis for miR-182-5p expression in NSCLC. **a**. Forest plot for overall SMD; **b**. Subgroup analysis; **c**. Funnel plot of publication bias; **d**. Sensitivity analysis

**Fig. 7:**
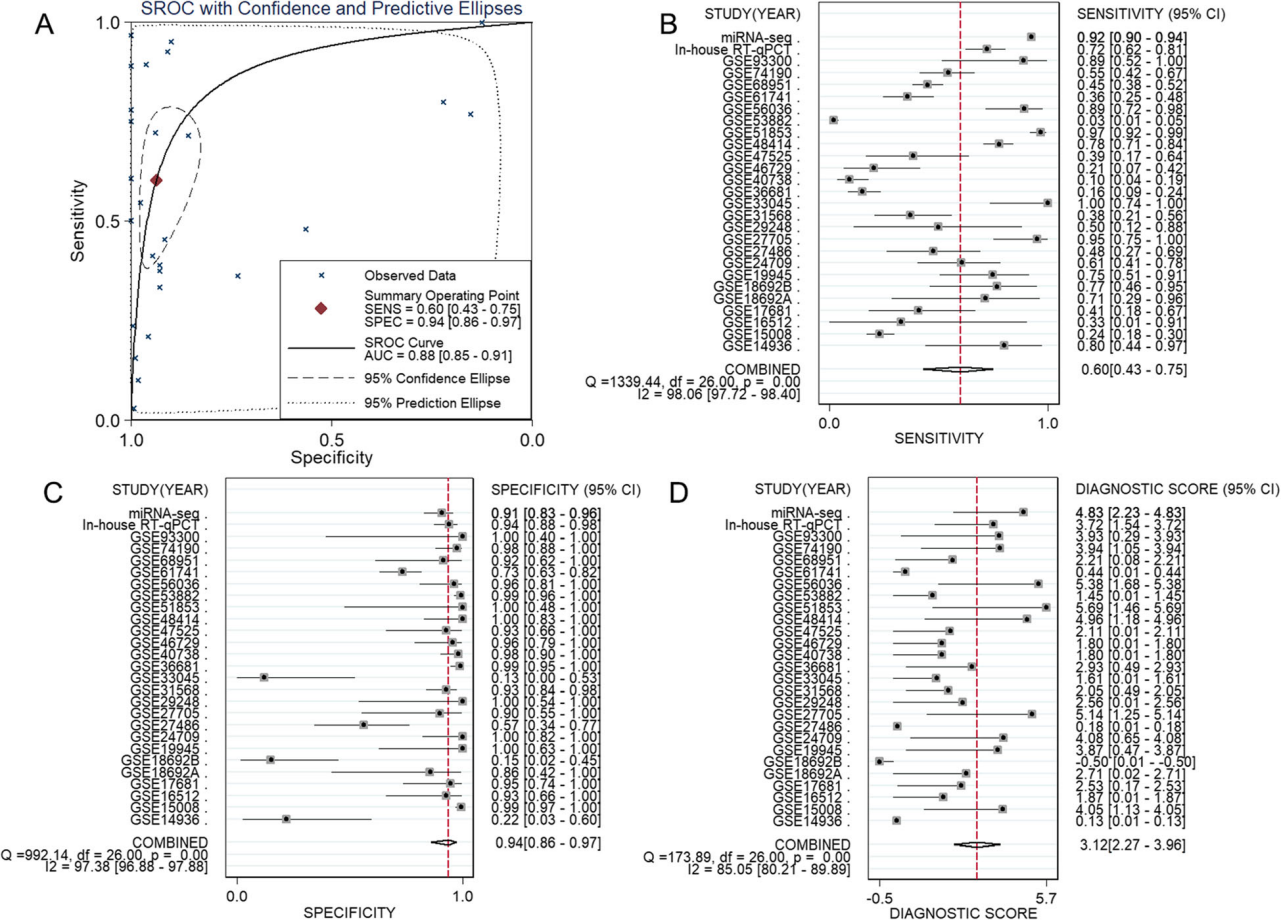
The distinguishing power of miR-182-5p in NSCLC tissues based on data from all studies. **a**. SROC curves; **b**. Forest plot for sensitivity; **c**. Forest plot for specificity; **d**. Summary of diagnostic scores

Based on the inclusion and exclusion criteria for the literature search, no studies were eligible for inclusion in the meta-analysis. Thus, the meta-analysis included 2564 NSCLC samples (LUAD, *n* = 899) and 1161 noncancer samples obtained from the in-house RT-qPCR, miRNA-seq, and miRNA-chip data analyses. The results of this comprehensive meta-analysis were consistent with those of the GEO meta-analysis, which confirmed upregulation of miR-182-5p in NSCLC tissues (SMD = 0.89 (0.55–1.22) Figs. 6). Upregulation of miR-182-5p expression was more apparent in the tissue samples than in the serum samples, and miR-182-5p expression in the tissue samples had stronger discriminating power in terms of cancer versus noncancer than miR-182-5p expression did in serum samples (Figs. 3, 4, 5, 6 and 7).

### Molecular mechanism of miR-182-5p in NSCLC

#### Functional annotation of candidate target genes in a PPI network

**Fig. 8:**
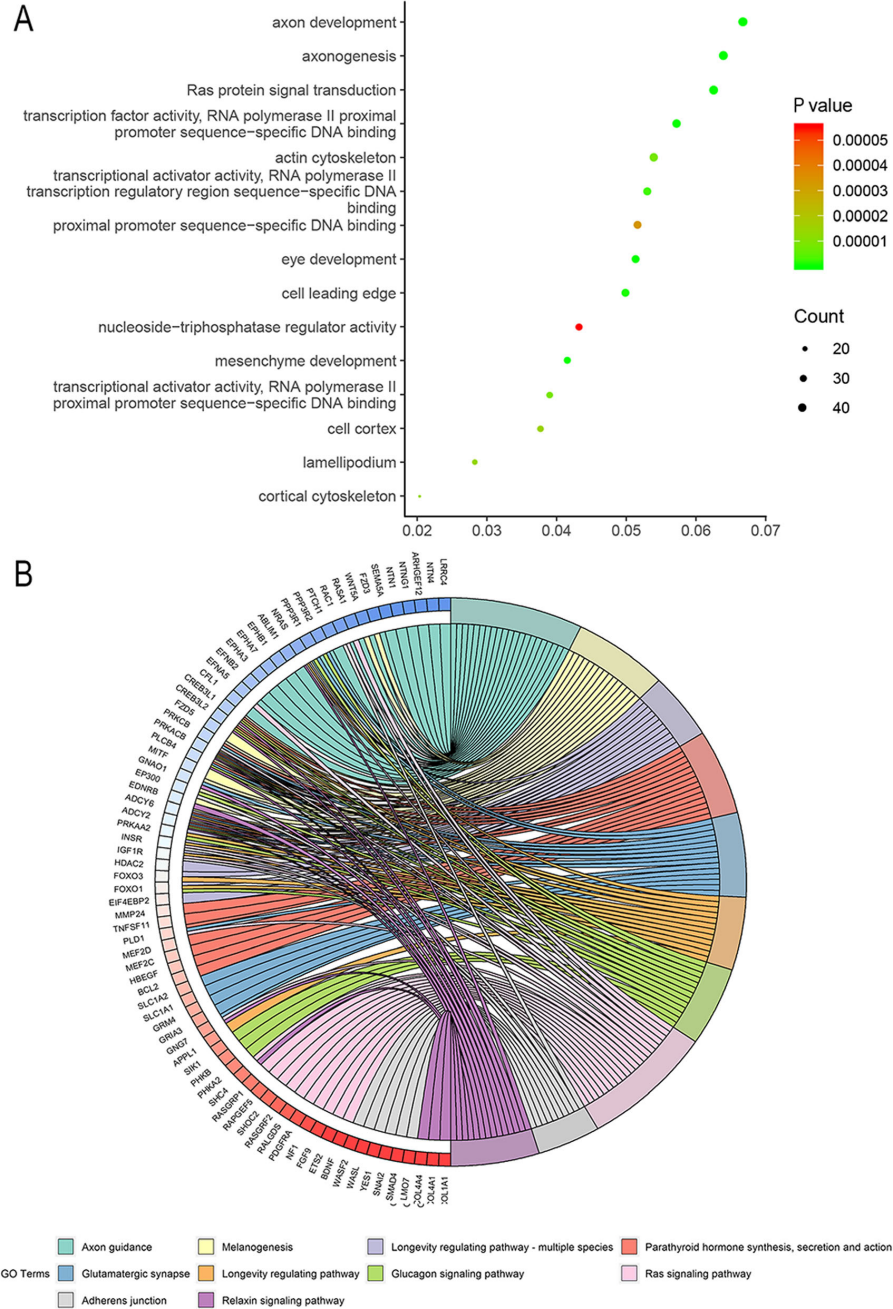
Functional enrichment analysis for candidate target genes of miR-182-5p. **a**. Bubble plot for gene ontology enrichment; **b**. Chord plot for Kyoto Encyclopedia of Genes and Genomes pathway analysis

**Fig. 9:**
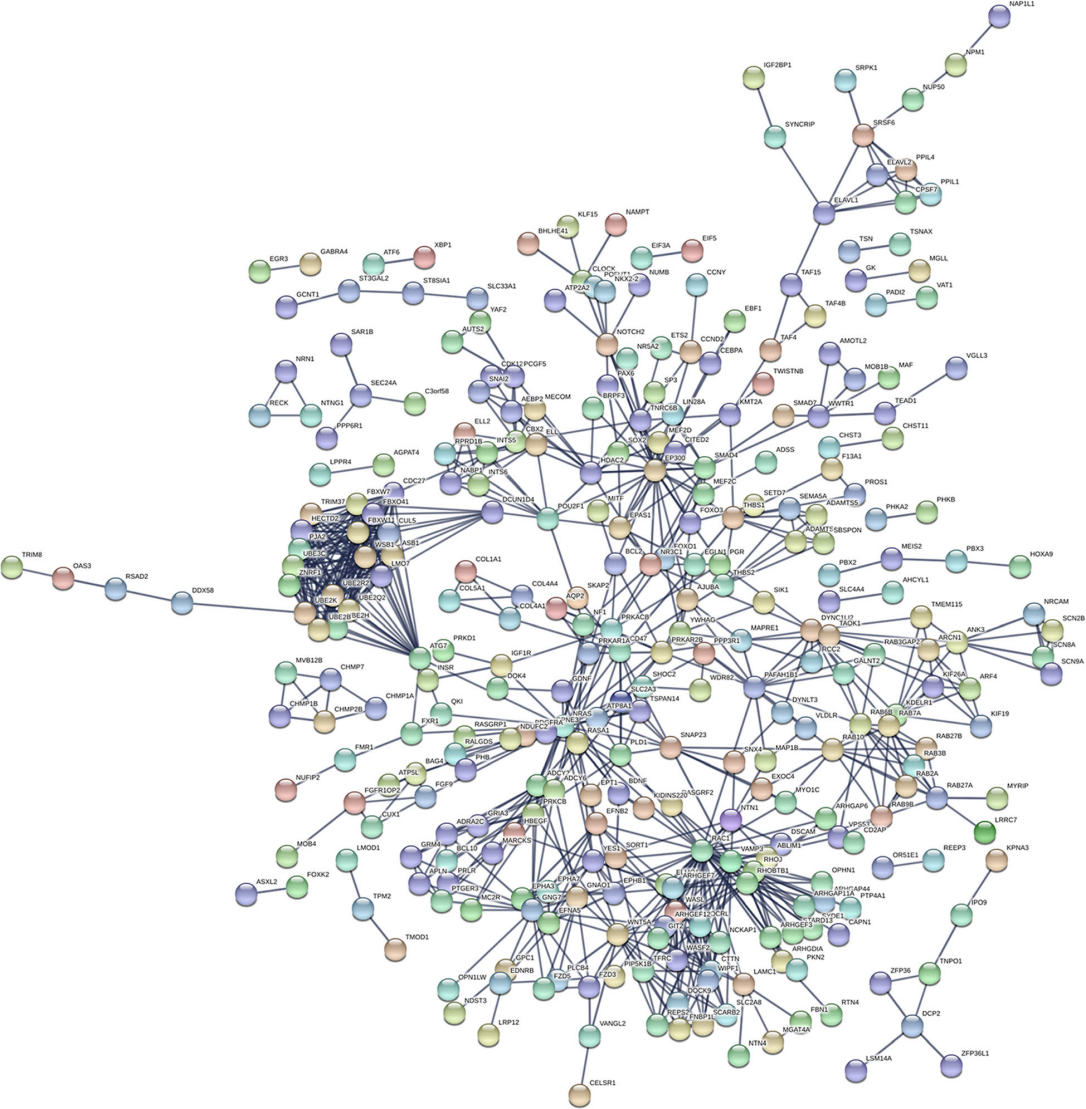
PPI network for candidate target genes of miR-182-5p. Nodes and strings in the network represented target genes and interactions between target genes

In total, 774 genes were identified as candidate target genes in eight of the 12 prediction platforms (Additional file 7). As shown in Fig. 8 and Table 6, these candidate target genes were significantly enriched in biological processes, such as axonogenesis, axonal development, and Ras protein signal transduction. According to the chord plot in Fig. 8, these target genes appeared to mainly participate in pathways involved in axonal guidance, melanogenesis, and longevity regulation in multiple species. The complicated interactions between the candidate target genes were illustrated in a PPI network (Fig. 9).

**Table 6:** Gene Ontology (GO) and Kyoto Encyclopedia of Genes and Genomes (KEGG) pathway analysis of candidate target genes of miR-182-5p

Category	Item	Count	P-value
GO-BP	axonogenesis	46	< 0.001
GO-BP	axon development	48	< 0.001
GO-BP	Ras protein signal transduction	45	< 0.001
GO-BP	eye development	37	< 0.001
GO-BP	mesenchyme development	30	< 0.001
GO-CC	cell leading edge	37	< 0.001
GO-CC	actin cytoskeleton	40	0.002
GO-CC	cortical cytoskeleton	15	0.002
GO-CC	lamellipodium	21	0.002
GO-CC	cell cortex	28	0.002
GO-MF	transcription factor activity, RNA polymerase II proximal promoter sequence-specific DNA binding	41	< 0.001
GO-MF	transcriptional activator activity, RNA polymerase II transcription regulatory region sequence-specific DNA binding	38	< 0.001
GO-MF	transcriptional activator activity, RNA polymerase II proximal promoter sequence-specific DNA binding	28	< 0.001
GO-MF	proximal promoter sequence-specific DNA binding	37	< 0.001
GO-MF	nucleoside-triphosphatase regulator activity	31	< 0.001
KEGG	Axon guidance	21	< 0.001
KEGG	Melanogenesis	15	< 0.001
KEGG	Longevity regulating pathway - multiple species	11	< 0.001
KEGG	Parathyroid hormone synthesis, secretion and action	14	< 0.001
KEGG	Glutamatergic synapse	14	< 0.001

Note: BP: biological process; CC: cellular component; MF: molecular function

#### Validation of miR-182-5p targeting of HOXA9

**Fig. 10:**
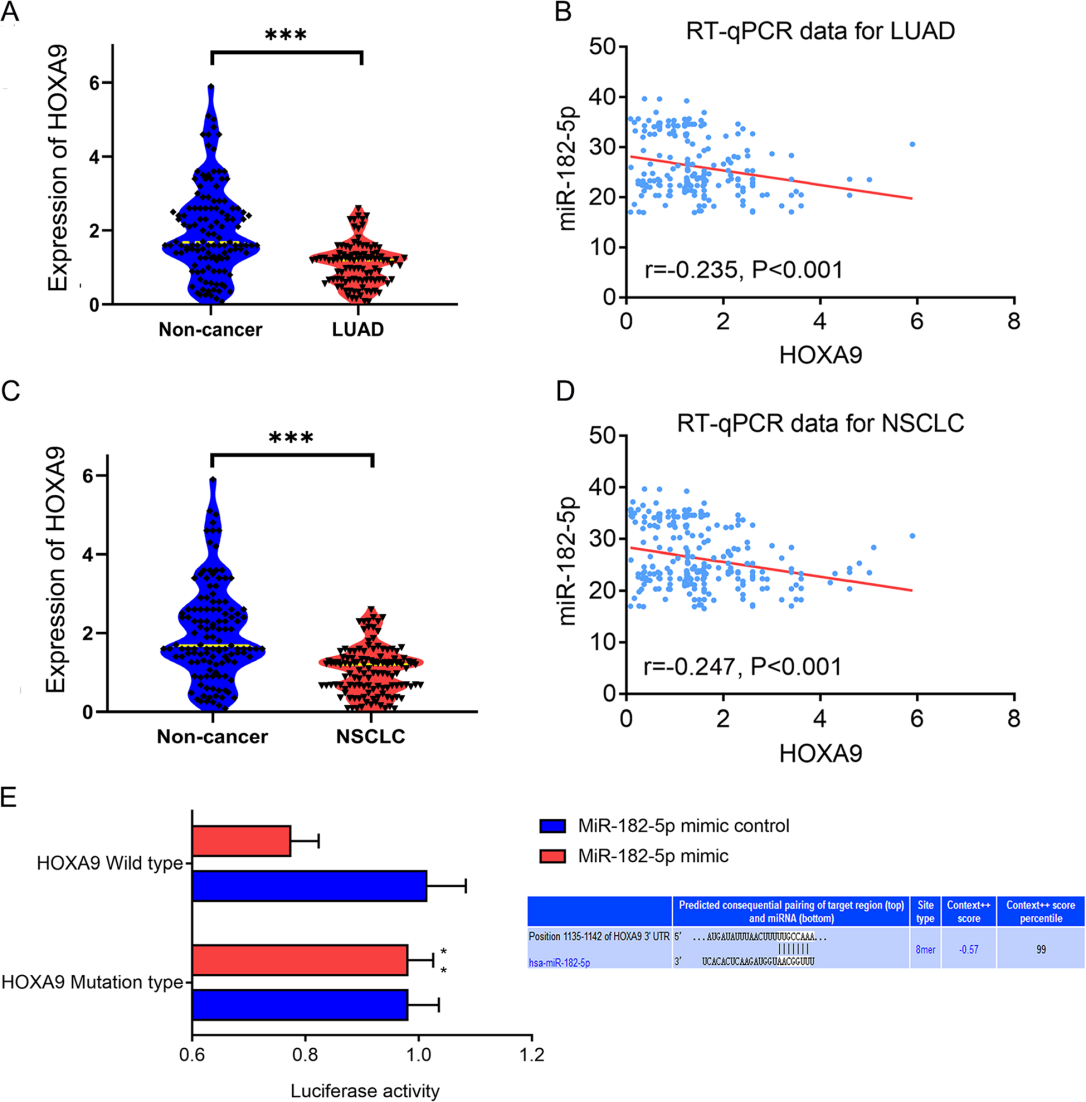
Validation of the targeting regulatory relationship between HOXA9 and miR-182-5p. **a**. Differential expression of HOXA9 in LUAD and noncancer tissues from RT-qPCR data; **b**. Correlation analysis based on in-house RT-qPCR data for miR-182-5p and HOXA9 expression in LUAD; **c**. Differential expression of HOXA9 in NSCLC and noncancer tissues from RT-qPCR data; **d**. Correlation analysis based on in-house RT-qPCR data for miR-182-5p and HOXA9 expression in NSCLC; **e**. Dual-luciferase reporter assay

Among the candidate target genes, we selected *HOXA9* and studied the relationship between it and miR-182-5p. As expected, *HOXA9* was downregulated in 101 LUAD tissue samples and all 125 NSCLC tissue samples, as shown by the RT-qPCR data (*P* < 0.001, Figs. 10A and C). Importantly, miR-182-5p expression was negatively correlated with *HOXA9* expression in 101 LUAD cases and 125 NSCLC cases (*r* = − 0.235, *r* = − 0.247, *P* < 0.001, Figs. 10B and D). The predictive binding sites for miR-182-5p in the 3′-UTR of *HOXA9* mRNA were imported from TargetScanHuman v.7.2. (Fig. 10E). According to a luciferase reporter assay, HEK-293 T cells co-transfected with psiCHECK-2/HOXA9 3′-UTR and miR-182-5p mimics showed significantly reduced luciferase activity as compared with that in a control group (*P* < 0.01, Fig. 10E).

## Discussion

MiRNAs are important in the occurrence and development of LC [43–48]. Recent studies reported that dysregulation of the expression of multiple miRNAs, including miR-182-5p, was significantly correlated with tumorigenesis of LC [34]. Although several studies have demonstrated the oncogenic effect of miR-182-5p in NSCLC [49–52], interactions between miR-182-5p and target genes in NSCLC remained unclear. In particular, the molecular mechanism of miR-182-5p in NSCLC was unclear.

We previously demonstrated the oncogenic consequences of miR-182-5p in LUSCs through a combinatory analysis of data from RT-qPCR assays of 23 samples obtained from LUSC patients treated in our hospital, miRNA-seq data, and miRNA-chip data. We hypothesized that the expression pattern of miR-182-5p was similar in all the subtypes of NSCLC. In the present study, miR-182-5p was overexpressed in LUADs according to data from RT-qPCR assays of 124 samples obtained from NSCLC patients treated in our hospital, miRNA-seq data, and miRNA-chip data. Thus, we investigated the clinicopathological significance of miR-182-5p in NSCLC using in-house RT-qPCR data, miRNA-seq data, miRNA-chip data, and data in the scientific literature to explore the underlying molecular mechanism via a functional analysis of target genes.

The aforementioned data supported marked upregulation of miR-182-5p in NSCLC. Furthermore, the results of the RT-qPCR assays supported the influence of upregulated miR-182-5p on malignant clinical progression of NSCLC, which was consistent with the findings of previous studies [49–52]. It should be noted that there were some contradictions between the results of the RT-qPCR assays and those of the miRNA-seq data analysis. The discord might stem from different sources of patient cohorts and methods for calculating miR-182-5p expression. The expression of miR-182-5p in the NSCLC samples analyzed using the in-house RT-qPCR was calculated based on the 2-Δcq algorithm In contrast, miR-182-5p expression in the miRNA-seq data was log2 (total_ reads per million + 1) transformed in IlluminaHiSeq_miRNASeq platform. Nevertheless, miR-182-5p was upregulated in both the LUAD and NSCLC cohorts according to the miRNA-seq data, and miR-182-5p exhibited a trend toward elevated expression in samples from patients with malignant clinical progression of NSCLC, which was in agreement with the overall results.

The SROC curves generated from all the datasets suggested that miR-182-5p could differentiate between LUAD or NSCLC and noncancer lung tissues. We believe that the large number of LC samples (NSCLC, *N* = 2564; non cancer, *N* = 1161) included in the present study support the findings.

To yield a deeper understanding of the molecular basis of the role of miR-182-5p in the carcinogenesis of NSCLC, we carried out functional annotations for candidate target genes and created a PPI network. The results indicated that miR-182-5p may exert an oncogenic influence on NSCLC via involvement in various biological processes, such as axonogenesis, axonal development, and Ras protein signal transduction, as well as in pathways including axonal guidance, melanogenesis, and longevity regulation in multiple species. The intricate regulatory network between the candidate target genes in the PPI network indicated that cooperation or antagonism between target genes may constitute an important link in the course of NSCLC.

Among the candidate target genes, *HOXA9*, a member of the HOX gene family, encodes a series of transcription factors with critical roles in cancer [53]. Previous research showed that *HOXA9* had oncogenic functions in hematologic cancers and anticancer effects in breast cancer and NSCLC [54, 55]. In this study, to shed light on the regulatory relationship between miR-182-5p and *HOXA9*, we studied the expression level of *HOXA9* in NSCLC and verified the relationships between miR-182-5p and *HOXA9* through a correlation analysis and dual-luciferase reporter assay. The results showed that upregulation of miR-182-5p in LUAD or NSCLC was significantly correlated with downregulation of HOXA9 in LUAD or NSCLC. The direct regulatory association between miR-182-5p and *HOXA9* was confirmed by the dual-luciferase reporter assay. Based on these findings, we conclude that miR-182-5p may affect the initiation and development of NSCLC by targeting *HOXA9* to diminish the tumor-inhibitory effect of *HOXA9* on NSCLC.

Several limitations of this study should be acknowledged. First, we did not validate the oncogenic effect of miR-182-5p on biological events of NSCLC through in vitro or in vivo experiments. Second, this study focused on the clinicopathological significance of miR-182-5p and the miR-182-5p-centered molecular mechanism in NSCLC. Alterations in the expression of various genes, such as *EGFR*, *ALK*, *ROS1*, *KRAS*, and *BRAF*, play essential roles in NSCLC, and these genes serve as targets of chemotherapy [56]. We did not explore the interactions between miR-182-5p and these genes in NSCLC. Third, the diagnostic value of miR-182-5p in serum was not verified in a large clinical NSCLC sample. Exosomal miRNAs have potential as diagnostic biomarkers for cancers because of their stability, nondegradability, and ease of detection [57]. The diagnostic value of exosomal miR-182-5p in NSCLC was not studied in current work.

## Conclusions

In conclusion, the oncogenic role of miR-182-5p in NSCLC was confirmed by comprehensively analyzing data obtained from RT-qPCR assays, miRNA-seq and miRNA-chip database. Multiple target genes, including *HOXA9*, may play a role in the molecular mechanism of *HOXA9* in NSCLC.

## Supplementary information

**Additional file 1: Figure S1.** Differential expression of miR-182-5p in LUAD and noncancer lung tissues based on data from in-house RT-qPCR, miRNA-seq and miRNA-chips. The distribution of miR-182-5p in LUAD and noncancer lung tissues was illustrated in the color of blue and red, respectively. A: GSE19945; B: GSE27486; C: GSE29248; D: GSE33045; E: GSE40738; F: GSE47525; G: GSE48414; H: GSE56036; I: GSE93300; J: GSE51853; K: in-house RT-qPCR; L: miRNA-seq

**Additional file 2: Figure S2.** Differential expression of miR-182-5p in NSCLC and noncancer lung tissues based on data from 9 miRNA-chips, in-house RT-qPCR and miRNA-seq. The distribution of miR-182-5p in NSCLC and noncancer lung tissues was illustrated in the color of blue and red, respectively. A: GSE29248; B: GSE56036; C: GSE93300; D: GSE53882; E: GSE18692-GPL4717; F: GSE18692-GPL4718; G: GSE27705; H: GSE33045; I: GSE36881; J: in-house RT-qPCR; K: miRNA-seq

**Additional file 3: Figure S3.** ROC curves for distinguishing power of miR-182-5p in LUAD based on data from in-house RT-qPCR, miRNA-seq and miRNA-chips. AUC: area under curves. An AUC value ranging from 0.1–1 indicated the increasing distinguishing power of miR-182-5p in LUAD. A: GSE19945; B: GSE27486; C: GSE29248; D: GSE33045; E: GSE40738; F: GSE47525; G: GSE48414; H: 51853; I: GSE56036; J: GSE93300; K: miRNA-seq; L: in-house RT-qPCR

**Additional file 4: Figure S4.** ROC curves for distinguishing power of miR-182-5p in NSCLC based on data from 9 miRNA-chips, miRNA-seq and in-house RT-qPCR. AUC: area under curves. An AUC value ranging from 0.1–1 indicated the increasing distinguishing effect of miR-182-5p in NSCLC. A: GSE56036; B: GSE93300; C: GSE53882; D: GSE18692-GPL4717; E: GSE18692-GPL4718; F: GSE27705; G: GSE33045; H: GSE36881; I: GSE24709; J: miRNA-seq; K: in-house RT-qPCR.

**Additional file 5: Figure S5.** Differential expression of miR-182-5p in NSCLC and noncancer lung tissues based on data from 16 miRNA-chips. The distribution of miR-182-5p in NSCLC and noncancer lung tissues was illustrated in the color of blue and red, respectively. A: GSE16612; B: GSE17681; C: GSE27486; D: GSE31668; E: GSE40738; F: GSE46729; G: GSE61741; H: GSE19945; I: GSE68951; J: GSE14936; K: GSE15008; L: GSE74190; M: GSE47525; N: GSE48414; O: GSE51853; P: GSE24709.

**Additional file 6: Figure S6.** ROC curves for distinguishing power of miR-182-5p in NSCLC based on data from 16 miRNA-chips. AUC: area under curves. An AUC value ranging from 0.1–1 indicated the increasing distinguishing effect of miR-182-5p in NSCLC. A: GSE16512; B: GSE17681; C: GSE27486; D: GSE31568; E: GSE40738; F: GSE46729; G: GSE61741; H: GSE68951; I: GSE14936; J: GSE15008; K: GSE19945; L: GSE47525; M: GSE48414; N: GSE51853; O: GSE74190; P: GSE29248.

**Additional file 7: Table S1.** Predicted target genes of hsa-miR-182-5p from miRWalk database. Putative target genes of hsa-miR-182-5p were predicted by 12 algorithms within mRNA selected regions.

## Data Availability

The datasets generated and/or analysed during the current study are available in the TCGA (TCGA-LUAD and TCGA-LUSC) (https://portal.gdc.cancer.gov/), GEO (GSE14936, GSE15008, GSE16512, GSE17681, GSE18692 (GPL4717 and GPL4718), GSE19945, GSE24709, GSE27486, GSE27705, GSE29248, GSE31568, GSE33045, GSE36681, GSE40738, GSE46729, GSE47525, GSE48414, GSE51853, GSE53882, GSE56036, GSE61741, GSE68951, GSE74190 and GSE93300) (https://www.ncbi.nlm.nih.gov/gds/), miRWalk (has-miR-182-5p binding site predictions within 3′-UTR region) (http://zmf.umm.uni-heidelberg.de/apps/zmf/mirwalk2/) and TargetScanHuman (TargetScan_7.2_ENST00000396345.1_predicted_targeting_details) (http://www.targetscan.org/cgi-bin/targetscan/vert_72/view_gene.cgi?rs=ENST00000396345.1&taxid=9606&members=miR-182-5p&showcnc=0&shownc=0&subset=1). Raw data of predicted target genes of miR-182-5p from miRWalk database was included in Additional file 7.

## Abbreviations

AUC: area under the curve
CI: confidence interval
GEO: Gene Expression Omnibus
GO: Gene Ontology
HOXA9: homeobox A9
KEGG: Kyoto Encyclopedia of Genes and Genomes
miRNA: microRNA
NSCLC: non-small cell lung cancer
PPI: protein–protein interaction
RT-qPCR: real-time quantitative polymerase chain reaction
SMD: standard mean difference
SROC: summary receiver operating characteristic
SROC: summary receiver operating characteristic (SROC)
TCGA: The Cancer Genome Atlas
