# Supplementary figures and images for: MiR-182-5p and its target HOXA9 in non-small cell lung cancer: a clinical and in-silico exploration with the combination of RT-qPCR, miRNA-seq and miRNA-chip
Source: BMC Med Genomics. 2020 Jan 6;13:3. doi: 10.1186/s12920-019-0648-7 (PMC6945423; doi:10.1186/s12920-019-0648-7)

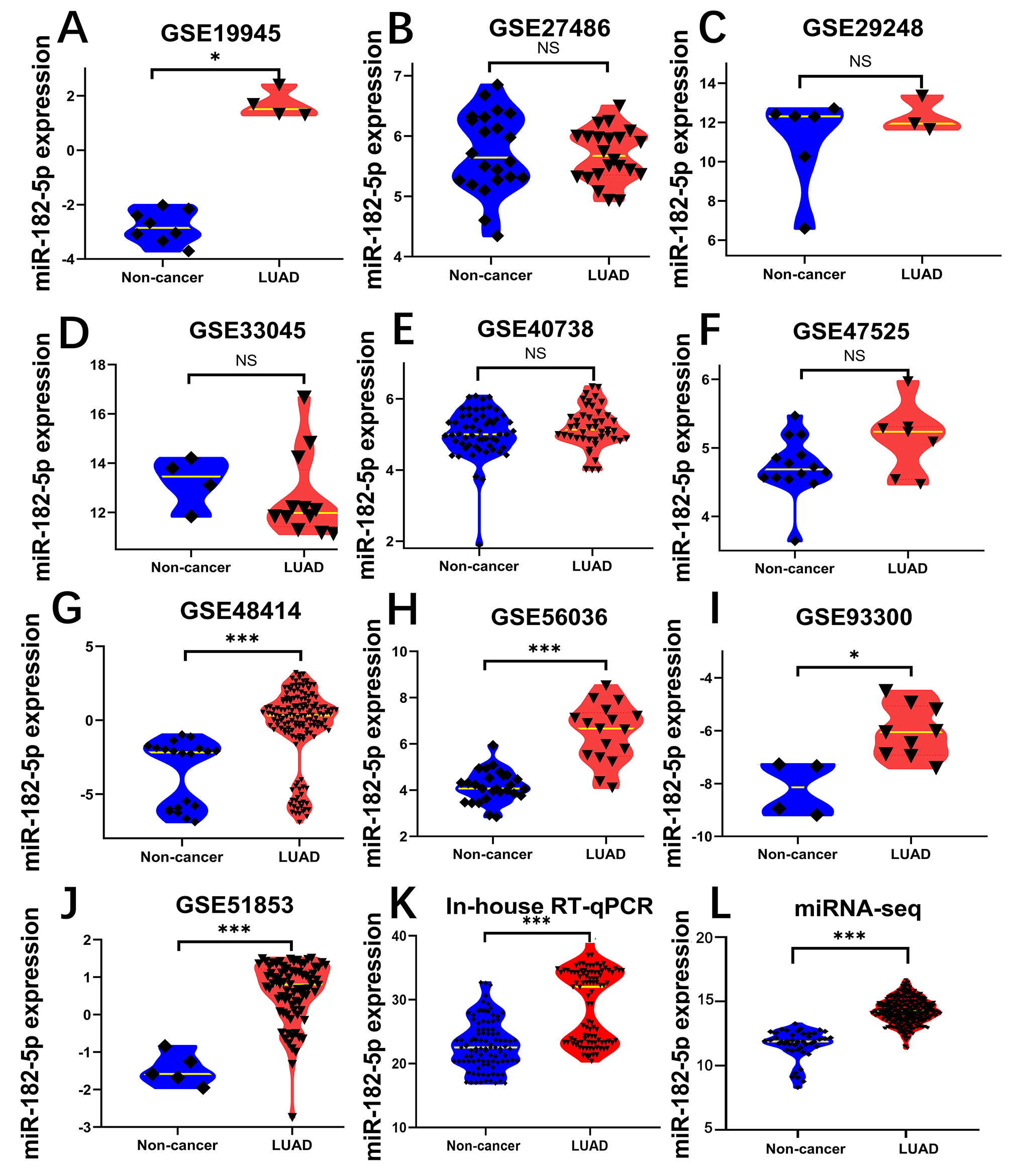

Supplement: Supplementary file 1 — Additional file 1: Figure S1. Differential expression of miR-182-5p in LUAD and noncancer lung tissues based on data from in-house RT-qPCR, miRNA-seq and miRNA-chips. The distribution of miR-182-5p in LUAD and noncancer lung tissues was illustrated in the color of blue and red, respectively. A: GSE19945; B: GSE27486; C: GSE29248; D: GSE33045; E: GSE40738; F: GSE47525; G: GSE48414; H: GSE56036; I: GSE93300; J: GSE51853; K: in-house RT-qPCR; L: miRNA-seq [file 12920_2019_648_MOESM1_ESM.tif]

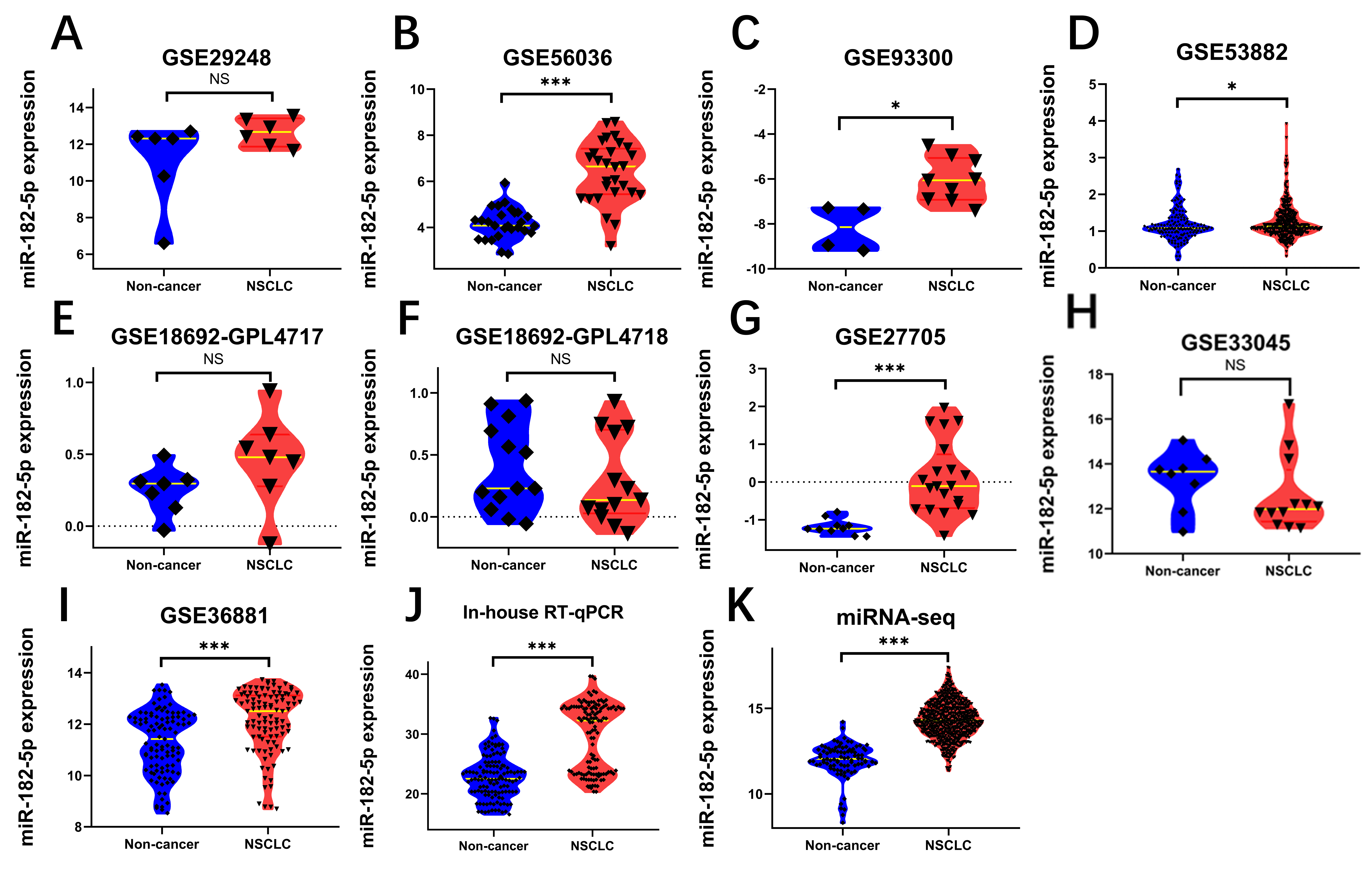

Supplement: Supplementary file 2 — Additional file 2: Figure S2. Differential expression of miR-182-5p in NSCLC and noncancer lung tissues based on data from 9 miRNA-chips, in-house RT-qPCR and miRNA-seq. The distribution of miR-182-5p in NSCLC and noncancer lung tissues was illustrated in the color of blue and red, respectively. A: GSE29248; B: GSE56036; C: GSE93300; D: GSE53882; E: GSE18692-GPL4717; F: GSE18692-GPL4718; G: GSE27705; H: GSE33045; I: GSE36881; J: in-house RT-qPCR; K: miRNA-seq [file 12920_2019_648_MOESM2_ESM.tif]

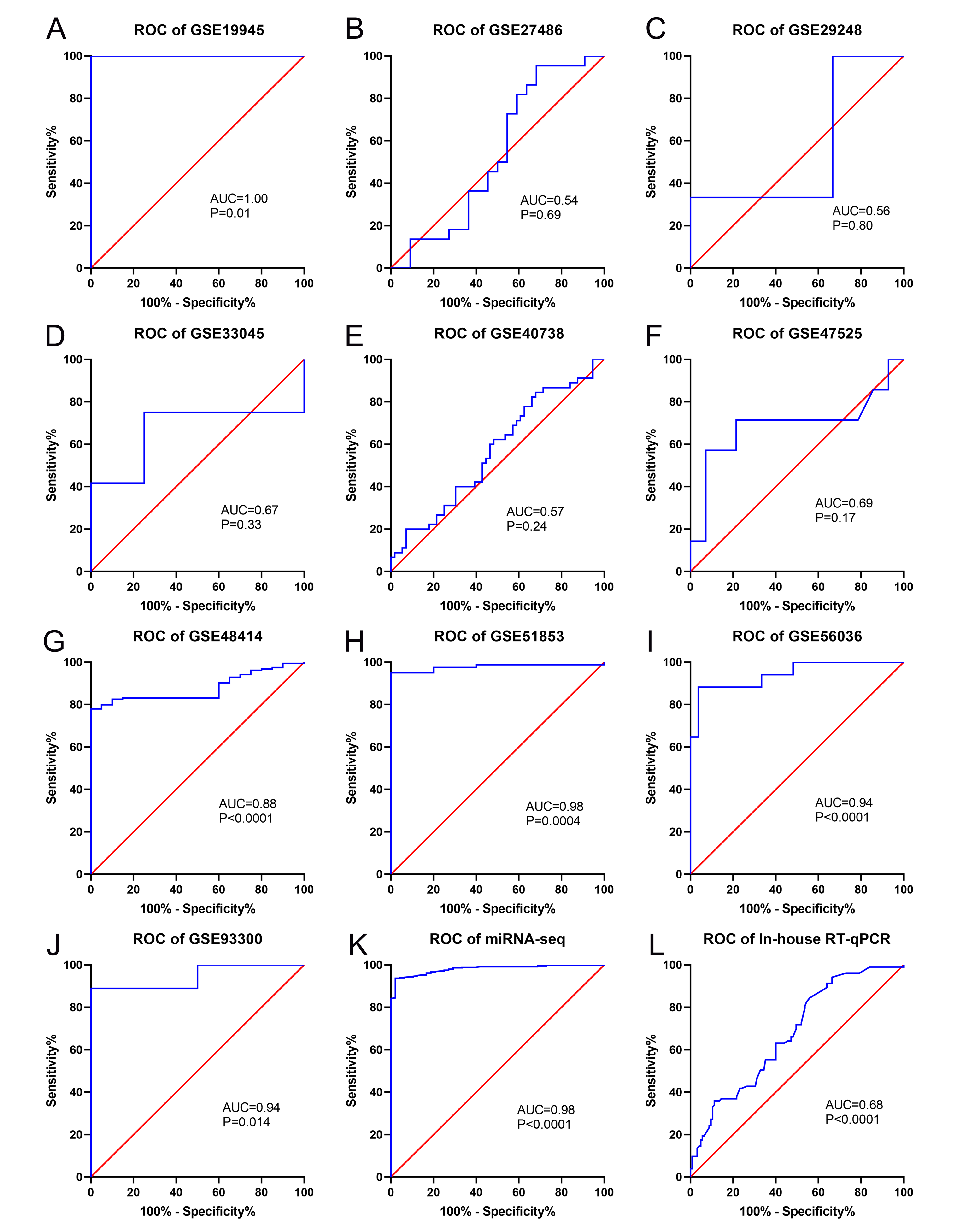

Supplement: Supplementary file 3 — Additional file 3: Figure S3. ROC curves for distinguishing power of miR-182-5p in LUAD based on data from in-house RT-qPCR, miRNA-seq and miRNA-chips. AUC: area under curves. An AUC value ranging from 0.1–1 indicated the increasing distinguishing power of miR-182-5p in LUAD. A: GSE19945; B: GSE27486; C: GSE29248; D: GSE33045; E: GSE40738; F: GSE47525; G: GSE48414; H: 51853; I: GSE56036; J: GSE93300; K: miRNA-seq; L: in-house RT-qPCR [file 12920_2019_648_MOESM3_ESM.tif]

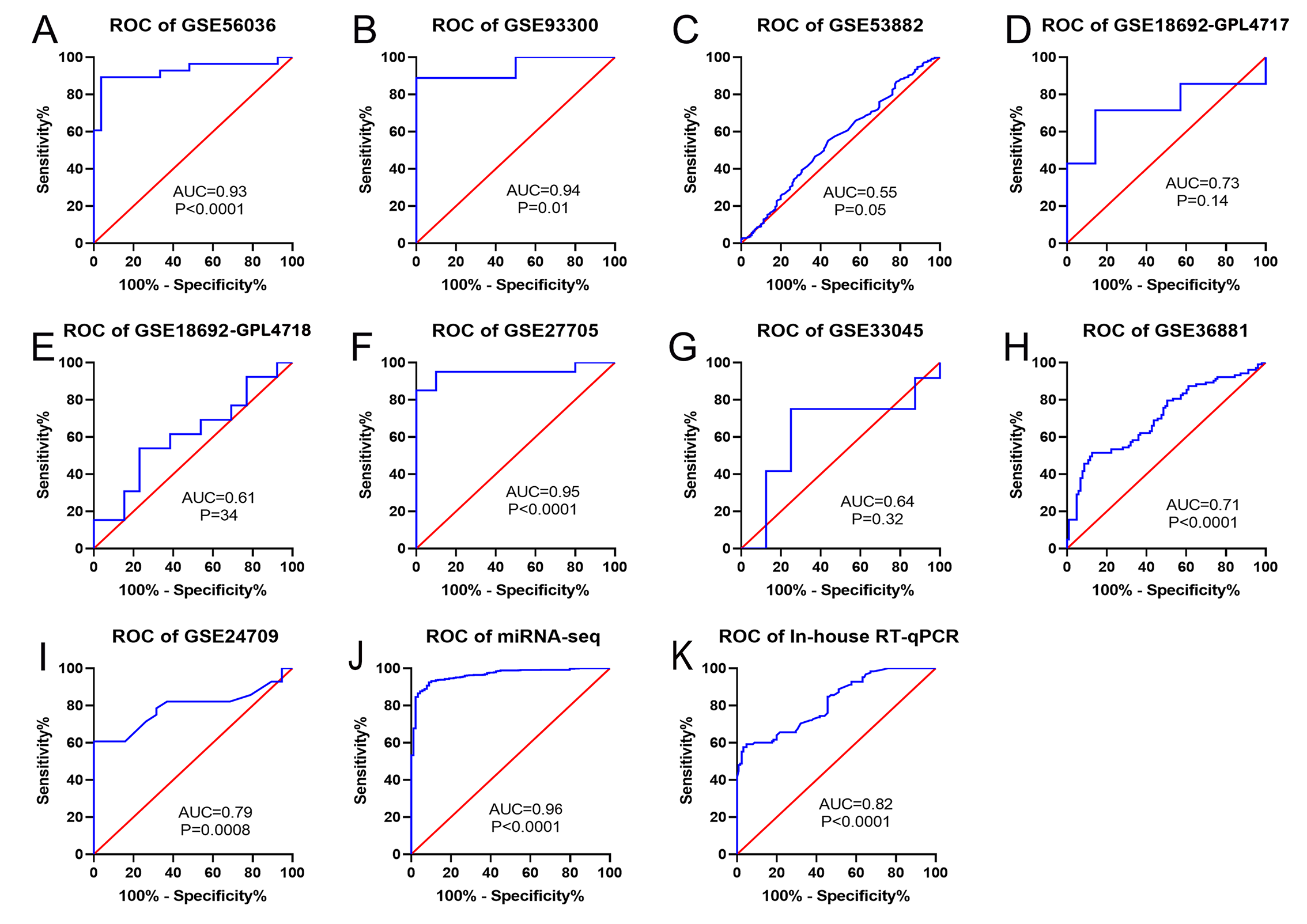

Supplement: Supplementary file 4 — Additional file 4: Figure S4. ROC curves for distinguishing power of miR-182-5p in NSCLC based on data from 9 miRNA-chips, miRNA-seq and in-house RT-qPCR. AUC: area under curves. An AUC value ranging from 0.1–1 indicated the increasing distinguishing effect of miR-182-5p in NSCLC. A: GSE56036; B: GSE93300; C: GSE53882; D: GSE18692-GPL4717; E: GSE18692-GPL4718; F: GSE27705; G: GSE33045; H: GSE36881; I: GSE24709; J: miRNA-seq; K: in-house RT-qPCR. [file 12920_2019_648_MOESM4_ESM.tif]

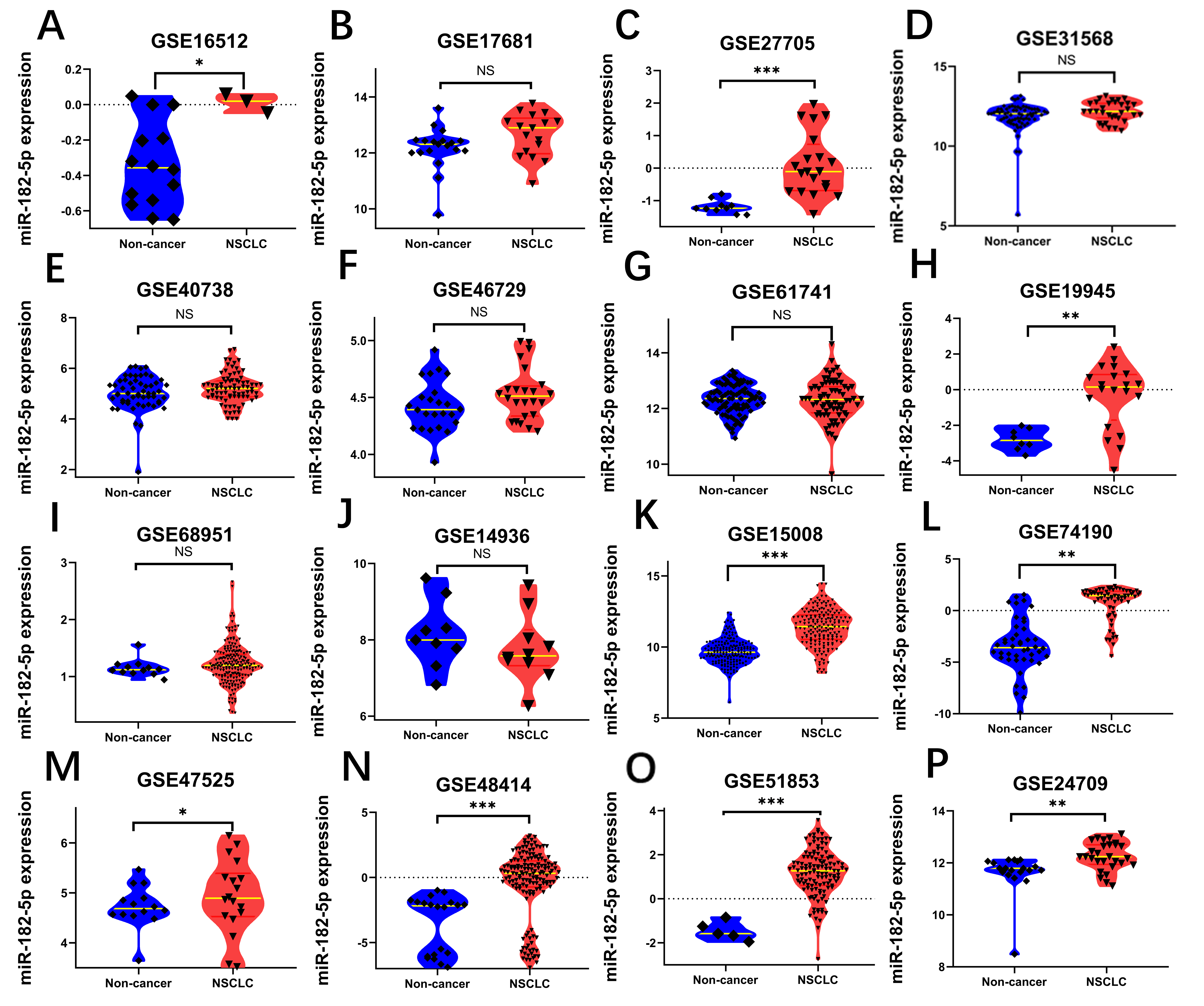

Supplement: Supplementary file 5 — Additional file 5: Figure S5. Differential expression of miR-182-5p in NSCLC and noncancer lung tissues based on data from 16 miRNA-chips. The distribution of miR-182-5p in NSCLC and noncancer lung tissues was illustrated in the color of blue and red, respectively. A: GSE16612; B: GSE17681; C: GSE27486; D: GSE31668; E: GSE40738; F: GSE46729; G: GSE61741; H: GSE19945; I: GSE68951; J: GSE14936; K: GSE15008; L: GSE74190; M: GSE47525; N: GSE48414; O: GSE51853; P: GSE24709. [file 12920_2019_648_MOESM5_ESM.tif]

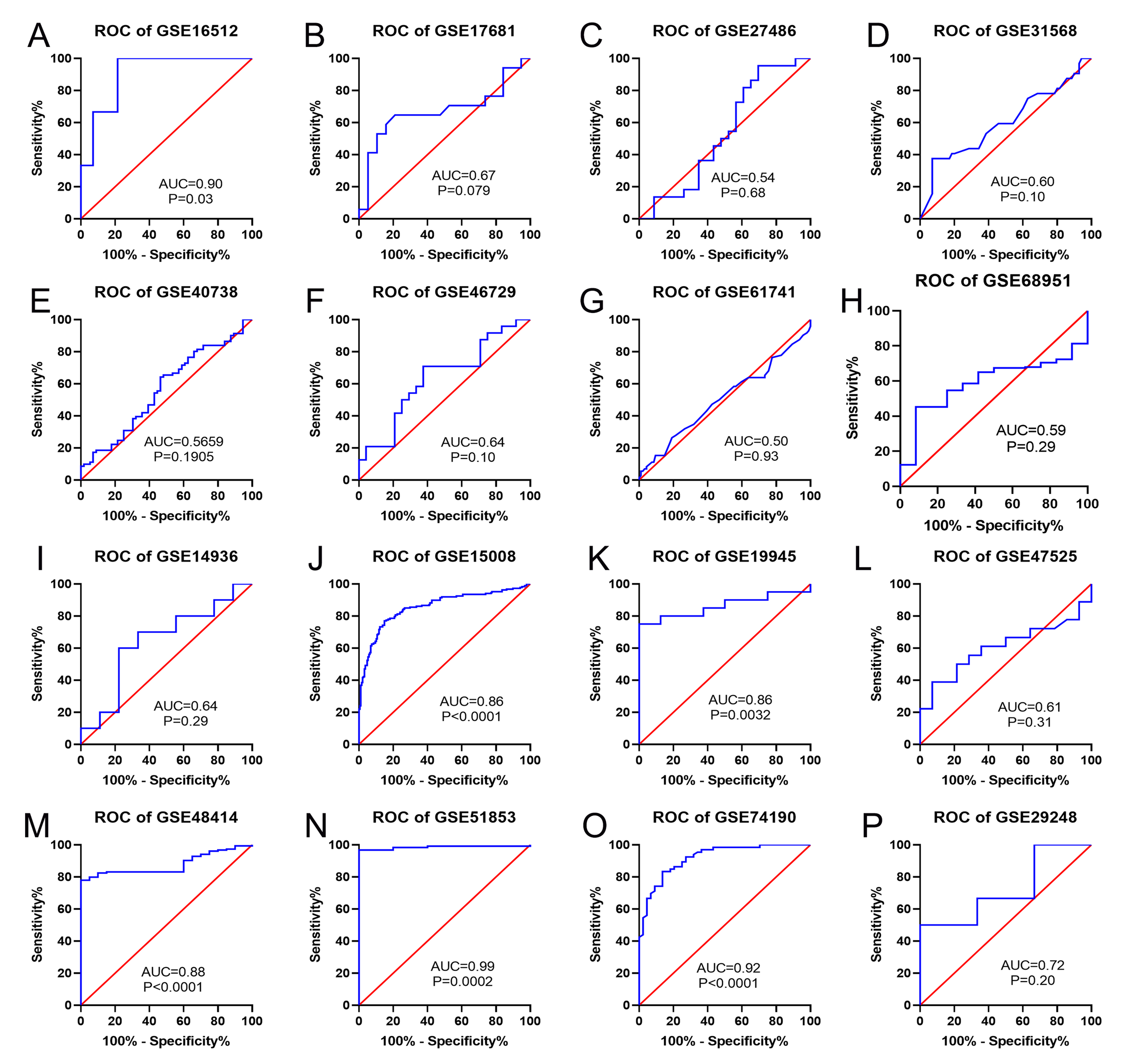

Supplement: Supplementary file 6 — Additional file 6: Figure S6. ROC curves for distinguishing power of miR-182-5p in NSCLC based on data from 16 miRNA-chips. AUC: area under curves. An AUC value ranging from 0.1–1 indicated the increasing distinguishing effect of miR-182-5p in NSCLC. A: GSE16512; B: GSE17681; C: GSE27486; D: GSE31568; E: GSE40738; F: GSE46729; G: GSE61741; H: GSE68951; I: GSE14936; J: GSE15008; K: GSE19945; L: GSE47525; M: GSE48414; N: GSE51853; O: GSE74190; P: GSE29248. [file 12920_2019_648_MOESM6_ESM.tif]
